# Pain: A Statistical Account

**DOI:** 10.1371/journal.pcbi.1005142

**Published:** 2017-01-12

**Authors:** Abby Tabor, Michael A. Thacker, G. Lorimer Moseley, Konrad P. Körding

**Affiliations:** 1 Centre for Pain Research, University of Bath, North East Somerset, United Kingdom; 2 Centre for Human and Aerospace Physiological Sciences/Pain Section, Neuroimaging, Institute of Psychiatry, Kings College London, London, United Kingdom; 3 Sansom Institute for Health Research, University of South Australia, Adelaide, South Australia, Australia; 4 Neuroscience Research Australia, Sydney, New South Wales, Australia; 5 Rehabilitation Institute of Chicago, Northwestern University, Chicago, Illinois, United States of America; Queen's University, CANADA

## Abstract

Perception is seen as a process that utilises partial and noisy information to construct a coherent understanding of the world. Here we argue that the experience of pain is no different; it is based on incomplete, multimodal information, which is used to estimate potential bodily threat. We outline a Bayesian inference model, incorporating the key components of cue combination, causal inference, and temporal integration, which highlights the statistical problems in everyday perception. It is from this platform that we are able to review the pain literature, providing evidence from experimental, acute, and persistent phenomena to demonstrate the advantages of adopting a statistical account in pain. Our probabilistic conceptualisation suggests a principles-based view of pain, explaining a broad range of experimental and clinical findings and making testable predictions.

## Introduction

In order to survive we must perceive our environment effectively, identify threats, and act to avoid damage to our body, or, if damage occurs, we must act rapidly to promote recovery. Pain is the fundamental experience associated with the perception of actual or potential damage to one’s self [1,2]. Despite its importance to human behaviour and to the human condition, little is known about its computational underpinnings.

During any kind of perception, humans can only rely on previous experiences and sensory information [3]. The information an individual can access, however, is almost always ambiguous, incomplete, or noisy [4–6]. As such, the way we perceive the world is often conceptualized in the perception literature as an act of statistically estimating the most likely properties of the world on the basis of noisy information [7,8]. In modern cognitive science, this is often formalized through statistical accounts, such as Bayesian inference [9–12], in which it is assumed that we perceive an estimation of our sensory signals based on current information and previous experience. This distinction between feed-forward sensory inputs and what the brain infers is central to most current theories of perception [13].

**Fig 1:**
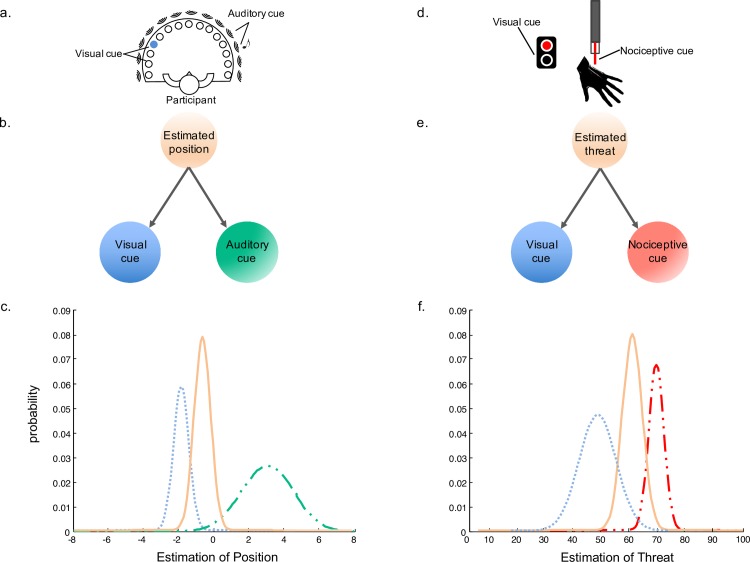
Cue combination in nonpain and pain perception are computationally equivalent. a–c) Simple models of cue combination involve combining two cues: in this case, the location of a visual stimulus and the location of an auditory stimulus. The influence of each cue is dependent on the noise or precision of the cue (probability distribution). The example shows the visual stimulus (dotted line) as more precise than the auditory cue (line-dot-dot); it, therefore, has more influence on the estimation of the object location (solid line). d–f) Combining cues related to the potential threat to one’s body promises to provide a better estimate of the overall threat. In this case, nociceptive information (line-dot-dot) is combined with visual information (dotted line) to produce an estimate of threat (solid line). In this example, nociceptive information is combined with a red visual cue, increasing the overall estimation of threat as compared to the combination of nociception and a blue cue, as demonstrated by Moseley and Arntz (2007).

There are strong mathematical models for the estimation of the state of the world. These models generally assume that each piece of information is statistically independent from the other, conditioned on the underlying variable that is estimated. For example, in the combination of auditory and visual information for localization (Fig 1A–1C), we may assume that each of the cues (*A*,*V*) is observed with noise (*σ*_*V*_ and *σ*_*A*_), a noisy measurement drawn from a Gaussian distribution relative to the true position (X):

$$A∼N(X,\sigma_{A}),V∼N(X,\sigma_{V})$$

In this case, according to Bayes rule, the optimal estimate ($\hat{X}$) is a weighted combination of the two:

$$\hat{X}=\frac{1/\sigma_{A}^{2}}{1/\sigma_{V}^{2}+1/\sigma_{A}^{2}}V+\frac{1/\sigma_{V}^{2}}{1/\sigma_{V}^{2}+1/\sigma_{A}^{2}}A$$

The model simply assumes that what we see and what we hear gets combined optimally to form an estimate of the world that considers not only different sources of information but also how noisy they are. The same equation can be used to model a broad range of multisensory phenomena [12].

### Pain as inference

That pain is also the result of a probability function based on incomplete or noisy information was mooted over 20 years ago and continues to inform clinical models of pain [14,15]. Recently, however, the idea has begun to gain traction in more theoretical and basic science fields, instigating the formalization of pain as an inferential process [16–19]. Relevant to this is the substantial advance in the understanding of the physiology of nociception, the wide array of contextual factors that have been shown to modulate pain and the increasingly tenuous relationship between pain and tissue damage that develops as pain persists [2].

There is now a compelling body of literature to support an inferential model of pain. During the experience of pain, just as during other perceptual experience, the brain makes inferences based on incomplete information. Specifically, the most common trigger of pain is a somatosensory barrage that includes, but is not limited to, activity in high threshold primary receptors (nociceptors) and their projections. Physiologically, nociceptive input is always accompanied by—indeed, preceded by—a wide array of non-nociceptive input triggered by other somatosensory receptors and a multisensory suite of event-related information [17]. This suite of information needs to be integrated with prior knowledge and over time, in order to calculate the experience that would most favourably serve the immediate objectives of the organism. A wealth of experimental evidence suggesting that any credible indication of threat to body tissue can increase pain and any credible indication of safety to body tissue can decrease pain [16,20–22] clearly points to the notion that pain results when the immediate objective of the organism is bodily protection. Importantly, even in highly controlled laboratory experiments, away from the “real world,” pain does not show an isomorphic relationship with the state of the tissues nor with nociceptive barrage [15].

Understanding the generation of hugely variable pain experiences is of great importance because pain is definitively unpleasant and disabling [23]; a poor understanding of pain will result in erroneous decisions about the cause of pain and, therefore, about the best course of action. Given that persistent pain is arguably one of the world’s most burdensome health conditions [24,25], the pursuit of better models with which to make sense of pain is imperative. Here we propose that a statistical account of pain as an inference process promises to lead to computational insights into the mechanisms of pain and advance our understanding of the huge variation in pain experiences between and within individuals.

For any phenomenon there can be a physiological interpretation and a normative interpretation, which are not mutually exclusive [26]. Here we start with the notion that the experience of pain can be modelled as a perceptual experience reflecting unconscious optimal estimates about the state of the world, which includes the body, and our best course of action within it. We then extend this model, in line with a Bayesian inference framework, allowing the description of a broad range of pain phenomena. Conventionally, the investigation into such pain experiences has been driven by dominant stimulus–response experimental models; here we argue that the same phenomena can be advantageously reconceptualised within a statistical model, as a special case of a generic perceptual inference process.

## Concepts

This review will consider the experience of pain from a Bayesian inference perspective, specifically drawing upon examples of experimental pain, acute pain, and persistent pain. In each case, the Bayesian concepts of cue combination, causal inference, and temporal inference will be applied to demonstrate the theoretical and practical implications of describing pain as an inference problem.

### Experimental pain: A cue combination problem

In pain research, understanding the generation of hugely variable pain experiences is of great importance, with conventional linear stimulus–response models deemed inadequate. A broad range of experiments has now demonstrated that the same noxious stimulus produces a hugely variable pain response, even within the same individual, within a controlled laboratory environment [16,21,27–31]. Moseley and Arntz (2007) conducted an experiment to this effect, considering the role of context and implicit expectation on the experience of pain. Pairing noxious stimuli with visual cues that carried implicit meaning—a red light (semiotically linked with heat and danger) [32] and a blue light (semiotically linked with cool and safety)—the authors accounted for variable pain experiences. The principle finding of this study revealed that a noxious stimulus is perceived as hotter and more painful when it is paired with a red visual cue than when it is paired with a blue one—for some participants, the visual cue accounted for a doubling of pain intensity (Fig 1D and 1E).

Furthermore, compelling evidence shows cues can evoke pain even in the absence of a noxious stimulus. For example, healthy volunteers report pain during sham head stimulation according to the level displayed on the sham stimulator’s intensity setting [33]; clinical pain patients report pain in response to a visual stimulus, implying their painful limb has been touched when it has, in fact, not been touched [34]; and, in those with movement-evoked pain and swelling, the increase in both pain and swelling are exacerbated when the hand is made to look swollen during movement, even though the movements themselves are identical [35].

Prompted by such findings, it has been proposed that the experience of pain reflects an overall estimation relative to the amount of threat that is posed to the body in a particular environment [36,37], an estimation that requires the integration of relevant information from multisensory sources. Such a proposal demonstrates one of the core principles of Bayesian modelling: combining relevant cues generally provides a better estimate of the variable of interest [28–30]. For a cue combination tutorial, see Supporting Information.

Similar to the studies conducted in pain, typical experiments on Bayesian cue combination involve parametrically varying the reliability of two experimental variables. This could be the visual size and the haptic size [38] of an object. Similarly, it could involve varying the disparity between the position of a visual cue and the position of an auditory cue [39]. The literature accounts for many combinations of cues and clearly shows that humans are very good at combining information from multiple sources.

In accordance with Bayesian models of cue combination, typical pain experiments have uncovered an intuitive finding that the experience of pain is relative to, but not an absolute reflection of, nociceptive information [15]. This contradicts the dominant stimulus–response models of pain experimentation because it clearly demonstrates the potential potency of explicit and implicit cue combination.

### Placebo, nocebo, and causal inference

The notion that perceptual experience depends on the integration of information is a simplification of the real world readily exposed in experimental settings. In reality, we are constantly required to infer whether cues, from multiple sources including memories, expectations, and beliefs, belong together or whether they should be treated as separate. The problem of inferring whether cues belong together can be observed in the experience of pain. Being able to accurately differentiate between threatening and nonthreatening cues within one’s environment serves to protect on the one hand and advance on the other. Indeed, it could be argued that the very experience of pain is fundamentally dependent on the way in which pieces of information are either integrated or segregated.

**Fig 2:**
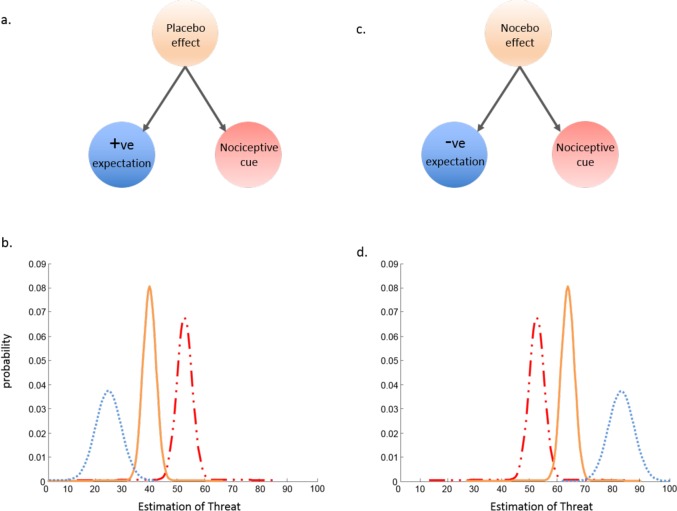
Causal inference in placebo and nocebo effects. The estimation of threat is determined by the relative integration of current information and prior expectations. Placebo effects (a–b) are associated with the relative weight of current sensory evidence, e.g., nociception (line-dot-dot), and expectations of safety, e.g., positive expectation associated with medication prescription (dotted line), resulting in an estimation of lowered overall threat (solid line). In contrast, nocebo effects (c–d) are associated with the relative precision (noise) of current sensory evidence (line-dot-dot) and expectations of harm, e.g., work-place demands (dotted line), resulting in an estimation of increased threat (solid line). The on-going estimations of relative safety and threat can be formulated as a casual inference problem, as demonstrated by Anchisi and Zanon, 2015.

Particularly relevant to this selective process are the diminished and enhanced pain experiences, which are considered under the banners of placebo and nocebo effects, respectively [40,41]. For example, taking a placebo pain relief tablet (Fig 2A and 2B) in association with a noxious stimulus can result in a reduction in the overall pain experience, even though the tablet does not contain pharmacologically viable compounds [42]. The aversive correlate of placebo, the nocebo effect, could also be conceptualized according to inference, whereby expectations of harm are integrated with noxious stimuli, increasing the overall threat that is estimated and the pain experienced [16,43,44]. Indeed, pairing a noxious cold stimulus with a red visual cue, which increases pain [21], could be labelled a nocebo effect.

The clearest experimental demonstration of Bayesian behaviour in pain perception is presented in a recent boundary-shifting study of causal inference [16], which has revealed the efficacy of Bayesian models to describe varying pain experiences in relation to the weighted integration of sensory information. Extending the work of Moseley and Arntz (2007), this investigation expands the scope of influencing factors affecting the experience of pain. Applying a full Bayesian Decision Model to a placebo conditioning paradigm, the authors were able to observe the intricate influence that prior expectations and current sensory evidence had on diminishing and enhancing pain experiences. Although their findings are complimentary to other results [31,45], their explanation differed. From a Bayesian position, the placebo effect occurs not because it is useful in and of itself, but rather because the underlying mechanism that determines the way in which information is combined or separated is useful for efficient, multisensory experience. As such, the broad range of factors captured by the umbrella terms placebo and nocebo can be considered perceptual illusions, the consequence of an optimal computational strategy.

Experiments in the pain field that typically test this hypothesis do so by manipulating the participant’s expectations associated with a sensory cue [31,46]. Many conditioning experiments that have used painful stimuli to condition fear have demonstrated selective generalization effects concordant with the idea of causal inference [47–49]. Anchisi and Zanon (2015) employed a conditioning protocol that exposed the bimodal nature of pain responses in accordance with a noxious cue and induced expectations [16]. However, the novel application of a full Bayesian Decision Model in this context revealed that both expectations (prior) and informational cues (likelihood) influenced pain, opening up the possibility that pain is influenced at different points of the inference process, up to and incorporating final decision-making. Indeed, recent discoveries using classical conditioning paradigms show that simultaneous pairing of benign somatosensory stimuli with noxious stimuli can quickly lead to reduced pain threshold and amplified pain on subsequent stimuli, clearly demonstrating an influence independent of expectation [50,51].

Applying Bayesian causal inference to the experience of pain can be seen as a natural progression from cue combination, setting out a tractable mathematical model that asserts the importance of accounting for different influences on the experience of pain. It offers a mathematical description of how different sources of information, whether sensory or nonsensory, are weighted in relation to each other. The greater the reliability of the information, the heavier the weight and, therefore, the more influence it has on experience [52]. This is relevant for a broad spectrum of pain experiences from everyday variation in pain given the same noxious input to the imprecise localization of pain that is observed in many persistent pain conditions [53]. It provides a pragmatic complexity for extending cue combination models of pain and deciphering the multimodal influences on the pain experience.

#### Experimental progression

Testing causal inference in a pain scenario in the laboratory would require several steps. Just like tests for cue combination, we would first need a conditioning phase to establish cues that are actually combined. Following the conditioning phase, participants could be presented with two cues that are incongruent with the pairings established in the conditioning phase. For example, a red colour cue may be presented with a temperature of 41°C, whereas a blue colour cue may be presented with a temperature of 49°C. At each paired stimulus presentation, the participant is required to provide a numerical pain rating. In accordance with the principles of causal inference, increasing incongruence between the stimuli will lead to the inference that they are associated with separate causes; one would predict that colour will no longer influence pain if it is too dissimilar to the implied nociception.

### Persistent pain and temporal integration

Up to this point we have considered a static analysis of the experience of pain and how this maps directly to the mathematical principles of Bayesian inference. Yet, the information about the level of threat posed to the body does not simply reflect prior expectations and current evidence but also incorporates changing experience over time. In order to estimate the current level of threat when the world is uncertain, we need to continually update our estimations based on the consequences of our actions in a changing environment. The consequences of our actions, the potential costs and benefits, vary over time and contribute to the pain we experience.

**Fig 3:**
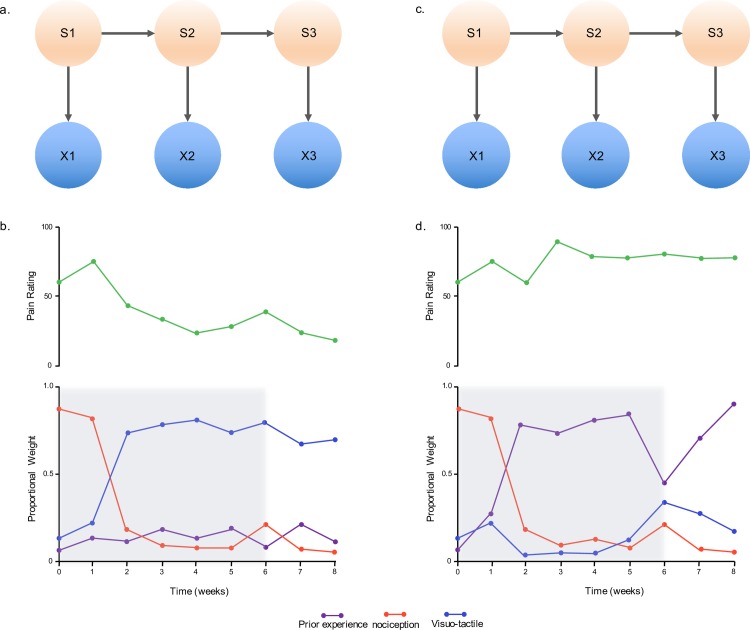
Information needs to be integrated over time. a–b) **Reduction in pain over time** (S1–S3). We estimate the level of threat posed to the body from noisy information, e.g., following wrist fracture and casting (x1–x3). In such cases, information is combined from past experiences (e.g., how the facture occurred, previous exposure to fracture), to form an optimal inference. If there is sufficient information available that reflects a decreasing level of bodily threat, an updated estimation of low threat will result, and the experience of pain will reduce over time. c–d) **Maintenance of pain over time**. During casting, visual, proprioceptive, and tactile information is restricted. In some cases, previous experiences may have greater influence on the estimation of threat as they are attributed more precision than the current sensory information, e.g., a highly traumatic incident that resulted in the fracture. In the absence of relevant safety cues, the estimation of threat may persist over time so that pain continues. Although an abstract example, this could be an alternative approach to understanding Complex Regional Pain Syndrome [53].

For example, take the experience of having an arm in an immobilizing cast after sustaining a fracture (Fig 3). Here we have access to visual information over time, but it is incomplete because the cast prevents full, active assessment of the limb. Consequently, the reliability of the information is reduced, and past experiences will be employed to improve the estimate of the state of the arm. Such a reliance on previous experience over current sensory evidence risks establishing an out-of-date analysis of the consequence of action, an elevation of the anticipated costs of action. If this impoverished sensory environment continues over time, then an estimate that the body remains under threat persists, resulting in the need for a protective action and the maintenance of pain (Fig 3B).

If we adopt the notion of temporal integration in line with statistical inference, Phantom Limb Pain can be observed as an extreme clinical example of ultimately restricted sensorimotor interaction over time [54]. In the absence of a limb, individuals are required to estimate the threat posed to them without the ability to specifically test that estimation. As such, multiple sources of information including, but not limited to, previous experience, current visual cues, personal implications, and social consequences will inform the overall estimation of threat. Without adequate safety cues, which enable the individual to alter this potentially heavily weighted estimation of threat, the experience of pain will persist [55]. Attempting to identify and challenge highly precise and thus heavily weighted information becomes an important factor for the investigation and treatment of all chronic pain conditions.

Investigations into the progression of pain over time typically focus on the relationship between action and the potential threat to body tissue. The application of learning theory to pain has highlighted the role of generalization in the manifestation of persistent pain [47,56]. The recent proposal of the Imprecision Hypothesis of persistent pain [17], grounded in classical conditioning theory, proposes that pain emerges in the presence of overly noisy sensory information. As such, the original pain experience becomes generalized over time to behaviours that were previously not painful, resulting in a decrease in the repertoire of pain-free activities.

#### Experimental progression

Even though a large body of research has outlined the impact that prior experiences and motivations have on pain [46,57–60], there are few studies that study the temporal integration of this information, and none, of which the authors are aware, that invoke a changing cost function. The main difficulty with experimentally testing the effects of temporal integration is that over relatively short timescales, necessary for the majority of lab experiments, adaptation effects occur. A stimulus, repeated over long time scales, may be perceived as progressively less painful, an effect that can be related to prediction [61,62]. Hence, to properly explore the effects of temporal integration in a lab setting, we need to minimize this adaptation effect. Switching stimulus locations would be one option, which would assume that pain locations are not static. Using pulsed noxious stimuli is another possibility.

An example design would take the following format. Let’s say we have a noxious temperature stimulator on each arm. We condition the participant by giving low pulses (e.g., every 30 seconds at 42°C) to the left arm and high pulses (e.g., every 30 seconds at 48°C) to the right arm. Afterwards we give identical pulses to both arms (e.g., 45°C). In accordance with the principles of temporal integration, previous experiences inform current experiences. If pain is temporally integrated across pulses, then the stimulus to the right arm should be perceived as more painful than the identical stimulus to the left arm.

## Open Avenues

The perceptual modelling literature appears to be relevant for pain research in many ways. It seems that approaching pain research from a computational perspective suggests a range of new experiments, interpretations of past experiments, as well as the ability to reappraise clinical pain conditions. While there are countless potential applications of ideas from Bayesian perception research, we want to highlight one of them in the following.

**Fig 4:**
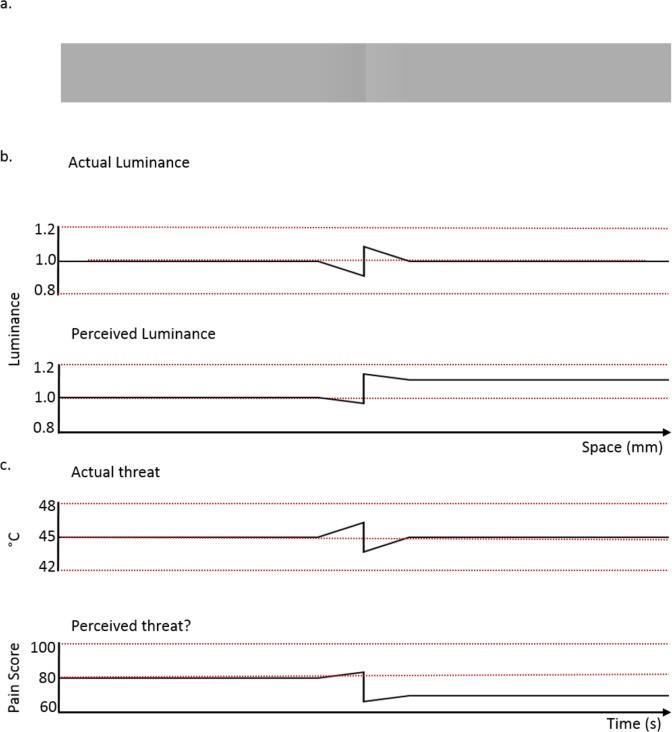
The Cornsweet illusion, a new hope for pain treatment? a) Two panels of equal luminance are presented; however, they are perceived to be different. Covering up the central portion where the panels meet will reveal that it is just in this section that the luminance ramps up and back down again, to give the illusion that the two panels are different. b) The actual stimulus luminance over space. c) Could a similar phenomenon exist in pain? By introducing a noxious experience that imperceptibly ramps up over time, jumps down, and then ramps back up to the original level imperceptibly to produce the illusion that the threat has decreased, thus lowering the experienced level of pain.

When we look at Fig 4A [63], we see two grey areas with the right one brighter than the left. What is remarkable is that the left and the right are actually equally bright—they are the same shade of grey. It is just that approaching the centre, the brightness slowly ramps down, quickly ramps up, and ramps down again to exactly the same level (4b). Somehow the brain misestimates the difference in brightness.

There are a number of explanations of the Cornsweet illusion [64,65], but a particularly simple one is that the brain assumes that brightness changes are either zero (consistent stimulus) or nonzero (varying stimulus). Such a mixture model is equivalent to causal inference, such that the brain misattributes a weak gradient to no gradient [66,67]. However, at the discontinuity in the middle, the gradient is so large that the misperception of the weak gradient is irrelevant. Hence, when integrating perceived luminance over space, we will make a mistake, which produces the illusion of a luminance change, resulting in the illusion of two different shades of grey.

Whatever the exact interpretation of the Cornsweet illusion, it provides an interesting conceptualization for increasing or decreasing the experience of pain. If pain perception is sufficiently analogous to visual perception, then conceivably, by introducing a noxious stimulus that slowly ramps up (imperceptibly), jumps down (strong relief!) and again slowly ramps up (again imperceptibly), it should be possible to produce the illusion of lowered pain (Fig 4C), or, more accurately, producing the percept that reflects the reduction of perceived threat of potential bodily damage and, therefore, lowering the experience of pain [62]. This example is not only contingent on the similar processing of visual and pain perception, but it also demands that the rules governing spatial perception (Cornsweet illusion) and temporal perception (pain illusion) are homogenous as well. The investigation of these conditions potentially provides mutual benefits for the fields of vision and pain. Yet, if we could produce the analogue of the Cornsweet illusion in pain research, it is possible we could produce the illusion of reducing the perceived threat of damage and the need for protection via pain. This, in turn, could be useful clinically.

## Concluding Remarks

The investigation into human experience from a perceptual inference perspective has provided insights into the potential mechanisms underlying those experiences. The application of such an approach to the investigation of the experience of pain could be highly interesting, as it promises to shed light on the computational underpinnings of pain. In addition, such an approach provides clear and testable hypotheses about the clinical progression of persistent pain, with the potential to expose new treatment approaches for the experience of pain.

## Supporting Information

S1 File Bayes tutorial. (PPT)

S2 File Bayes tutorial, notes. (DOC)

S1 Data Example datasets for Bayes tutorial, part I. Combining information from 52 neurons to decode movement.
(ZIP)

S2 Data Example datasets for Bayes tutorial, part II. Combining two cues in order to estimate the location of a target.
(ZIP)
