# Supplementary material for: Pain: A Statistical Account
Source: PLoS Comput Biol. 2017 Jan 12;13(1):e1005142. doi: 10.1371/journal.pcbi.1005142 (PMC5230746; doi:10.1371/journal.pcbi.1005142)
Supplement: S1 File — (PPT) [file pcbi.1005142.s001.ppt]

## Slide 1
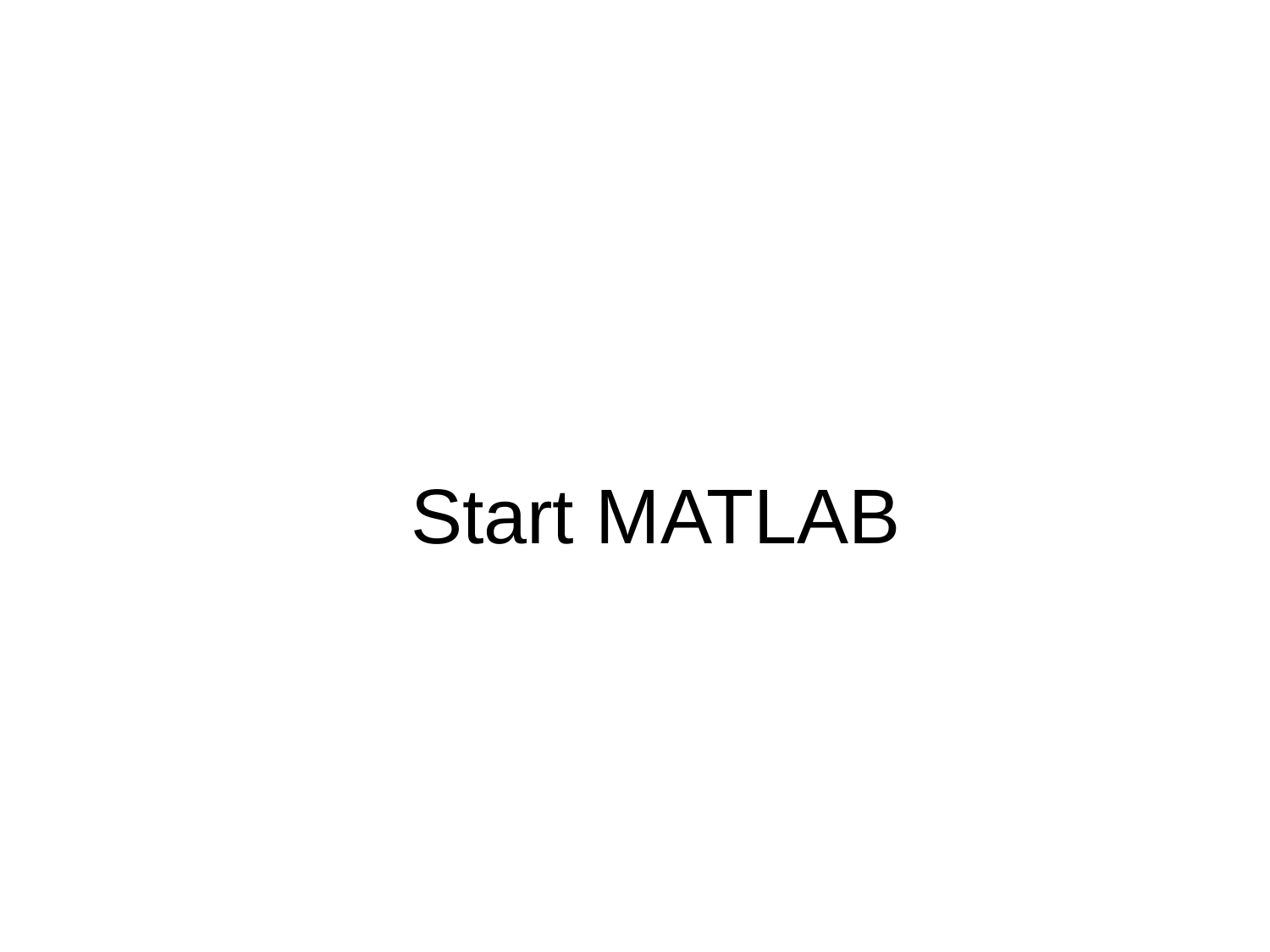

# Start MATLAB

## Slide 2
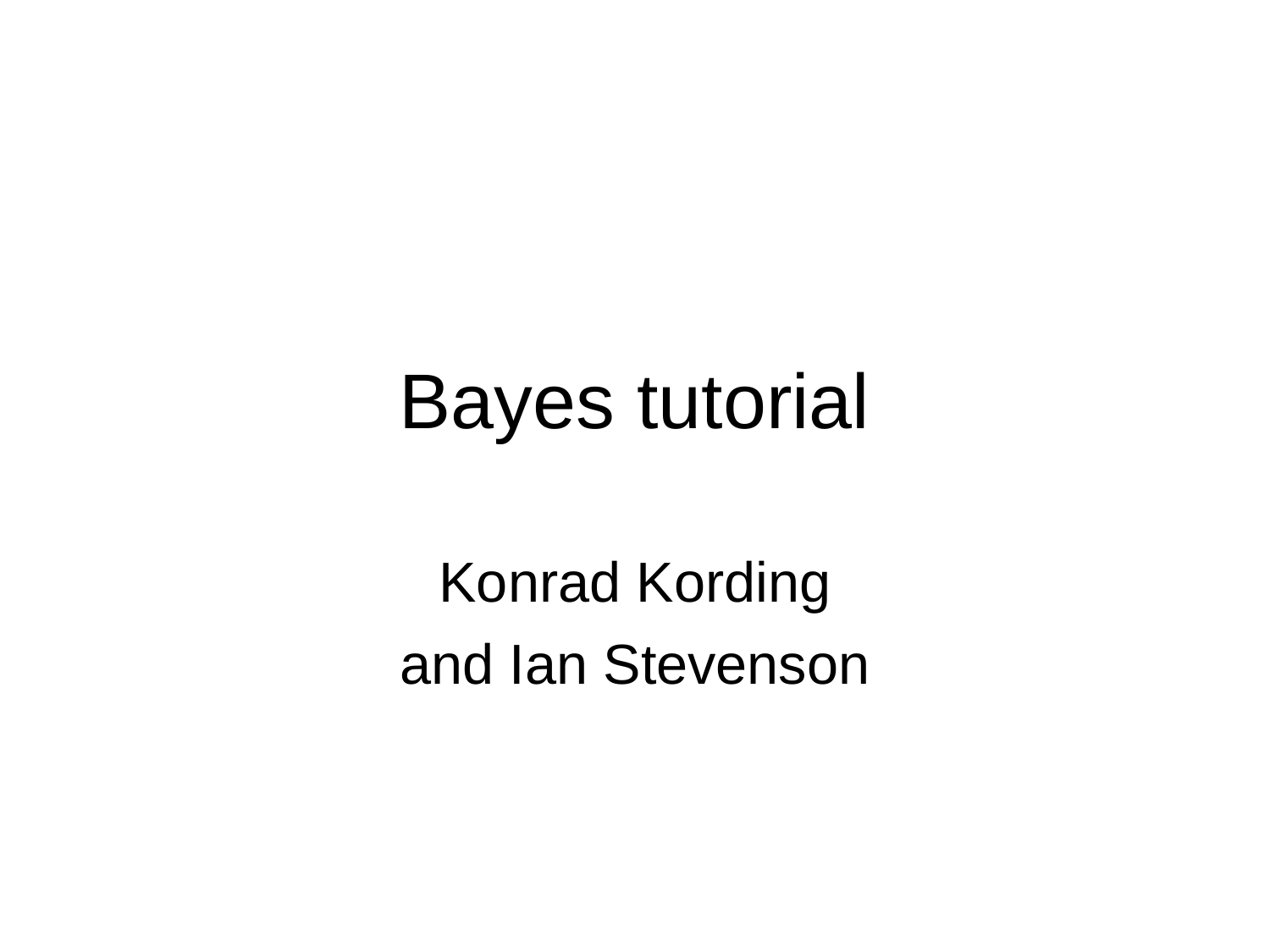

# Bayes tutorial
Konrad Kording
and Ian Stevenson

## Slide 3
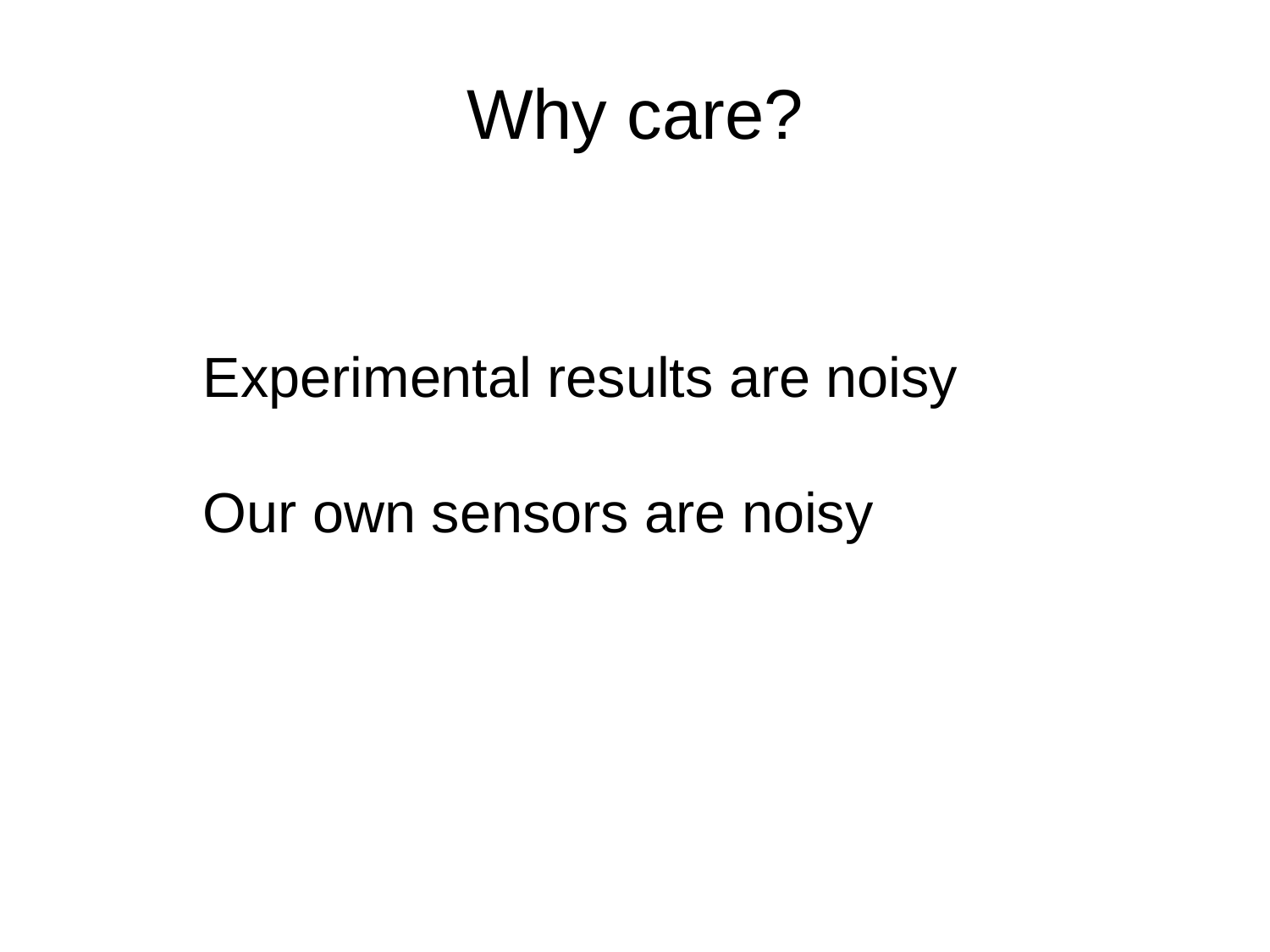

# Why care?
Experimental results are noisy
Our own sensors are noisy

## Slide 4
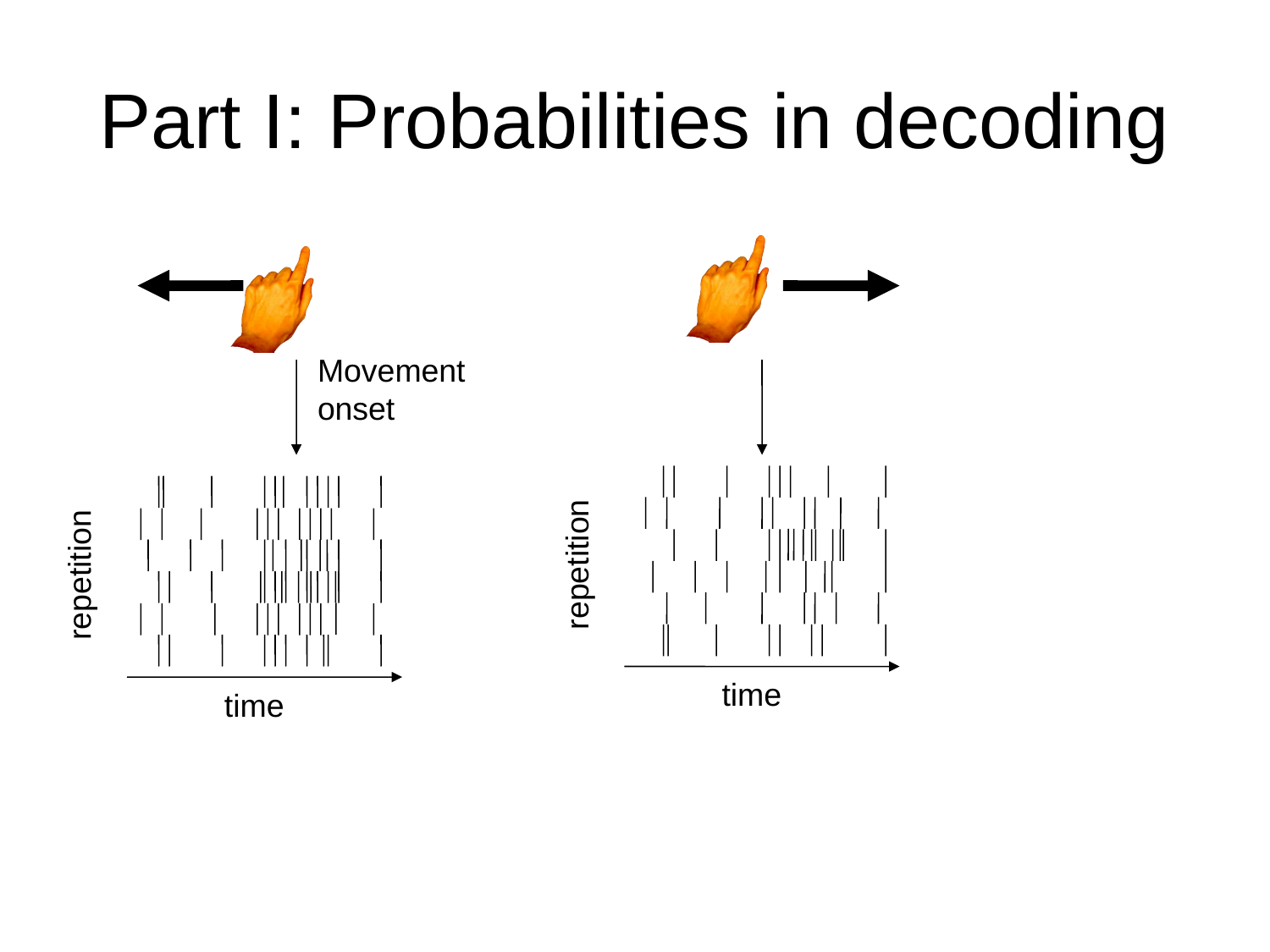

# Part I: Probabilities in decoding
Movement
onset
repetition
repetition
time
time

## Slide 5
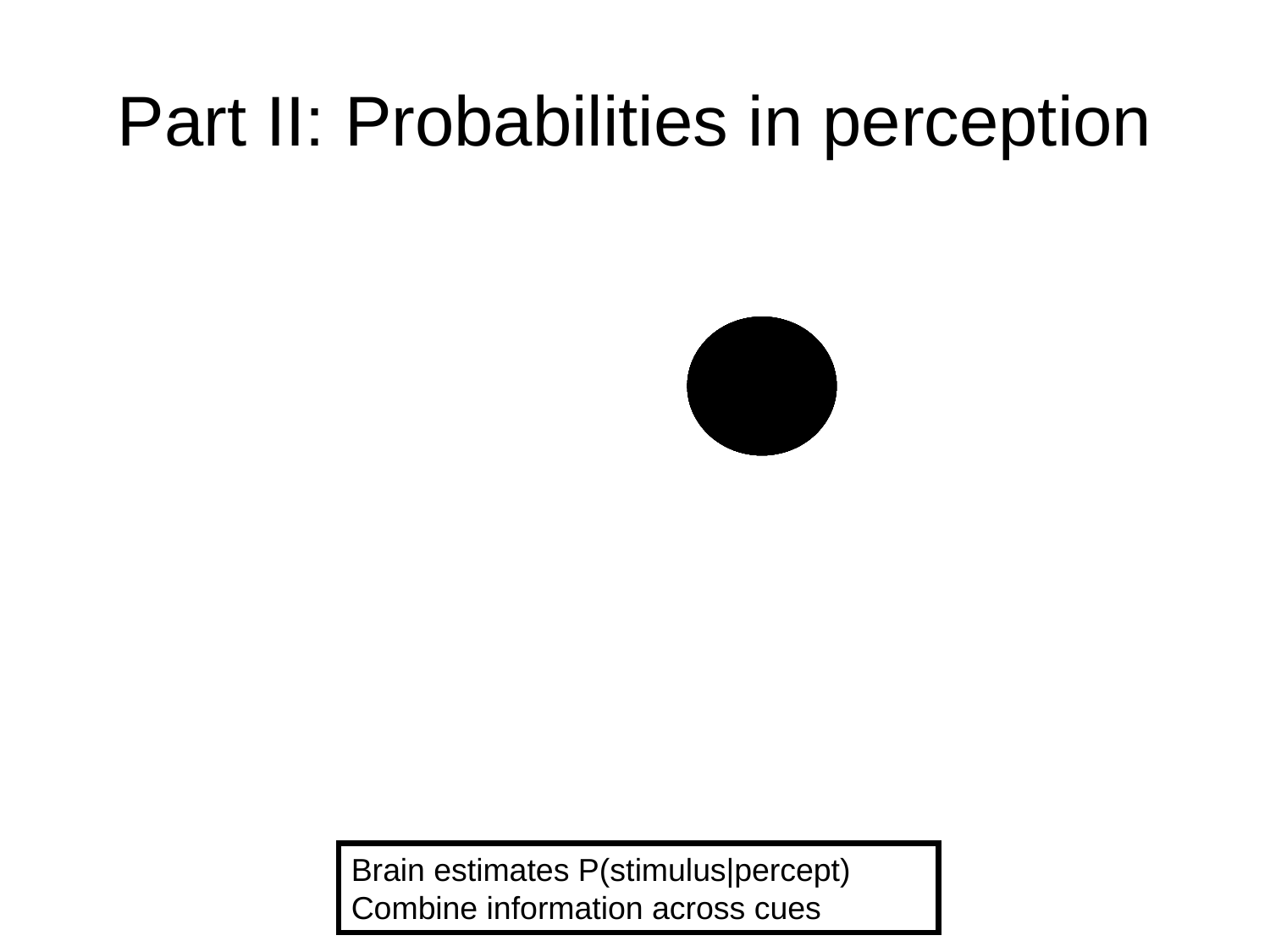

# Part II: Probabilities in perception
Brain estimates P(stimulus|percept)
Combine information across cues

## Slide 6
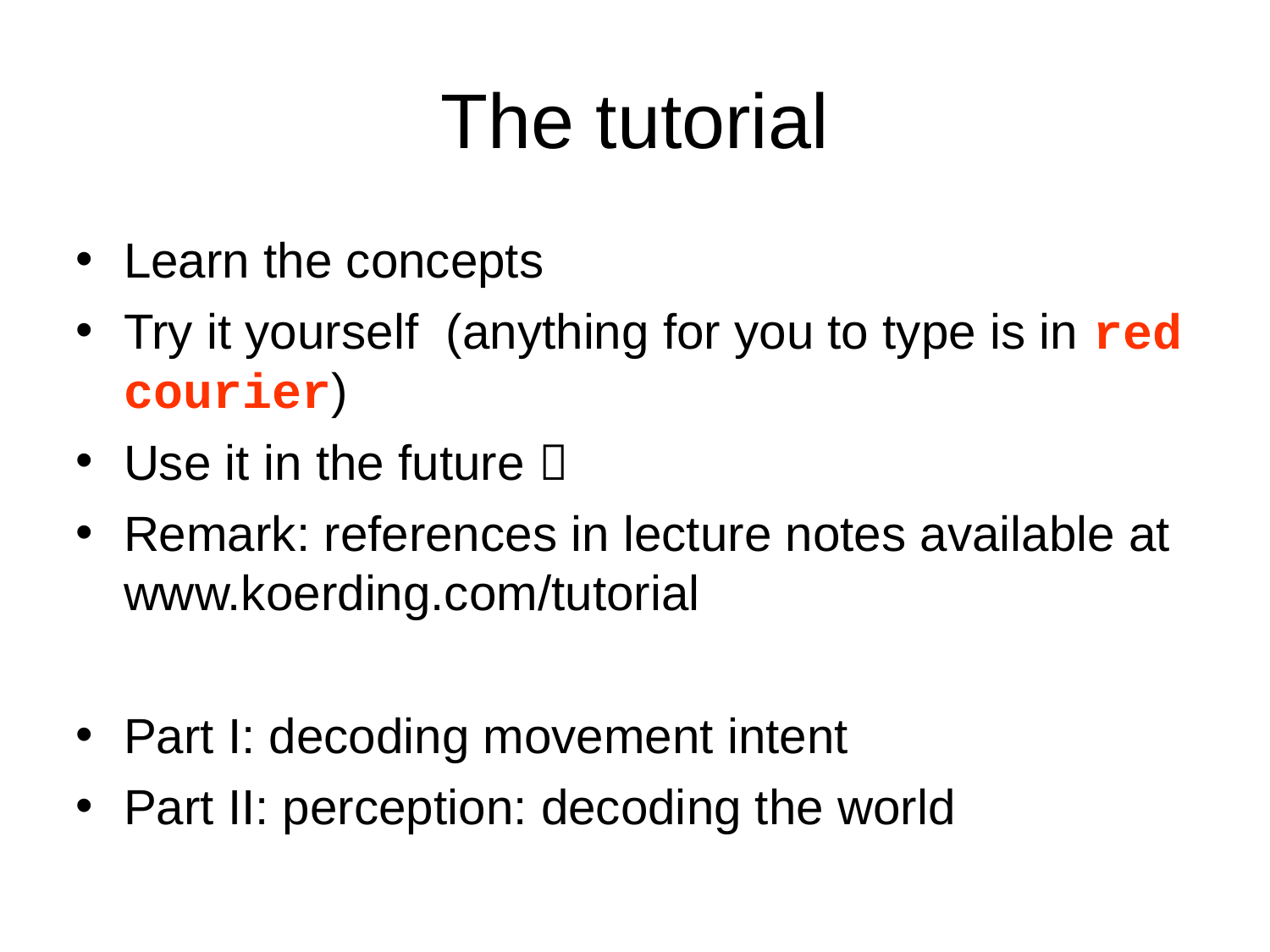

# The tutorial
Learn the concepts
Try it yourself (anything for you to type is in red courier)
Use it in the future 
Remark: references in lecture notes available at www.koerding.com/tutorial
Part I: decoding movement intent
Part II: perception: decoding the world

## Slide 7
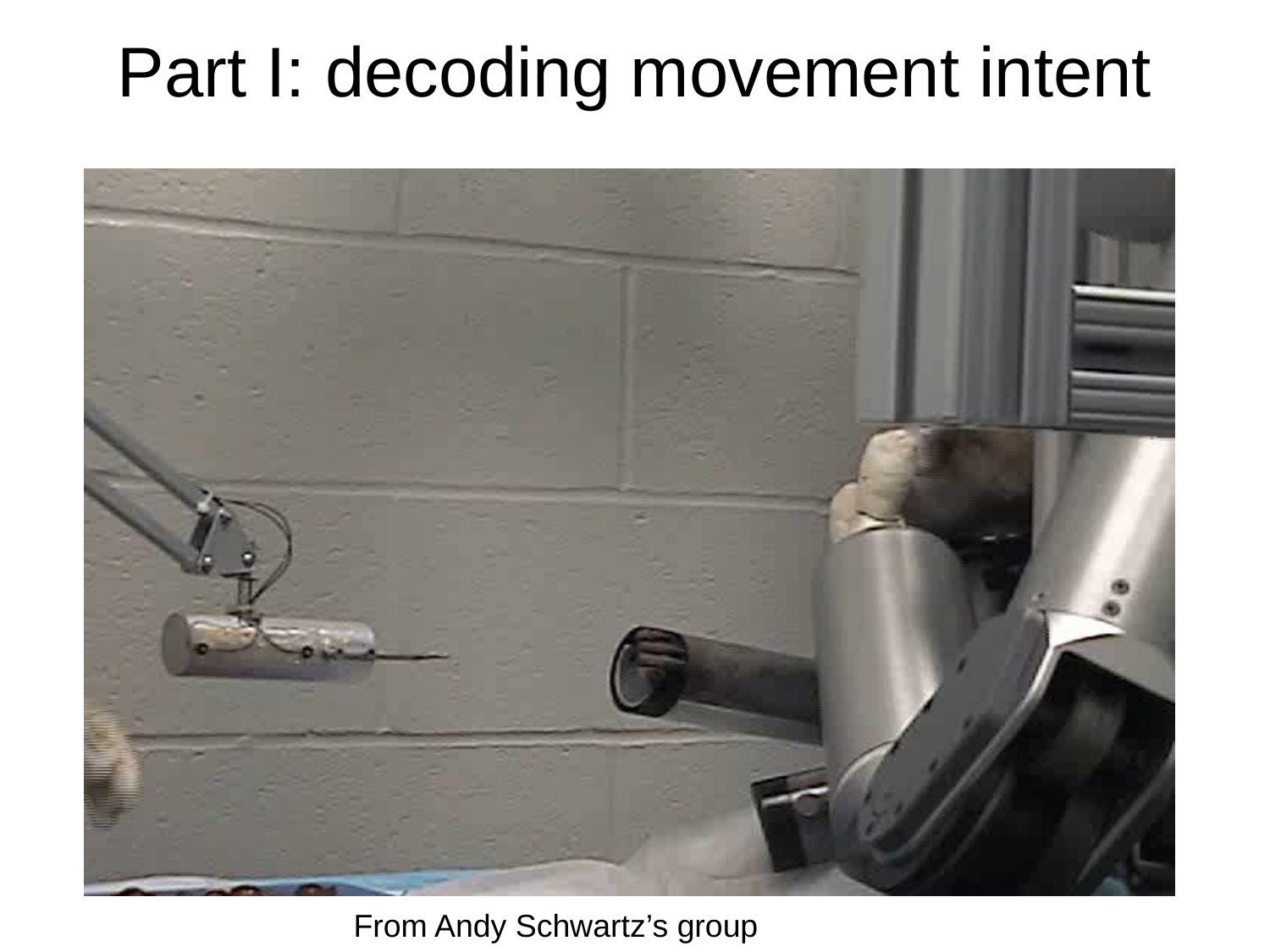

# Part I: decoding movement intent
From Andy Schwartz’s group

## Slide 8
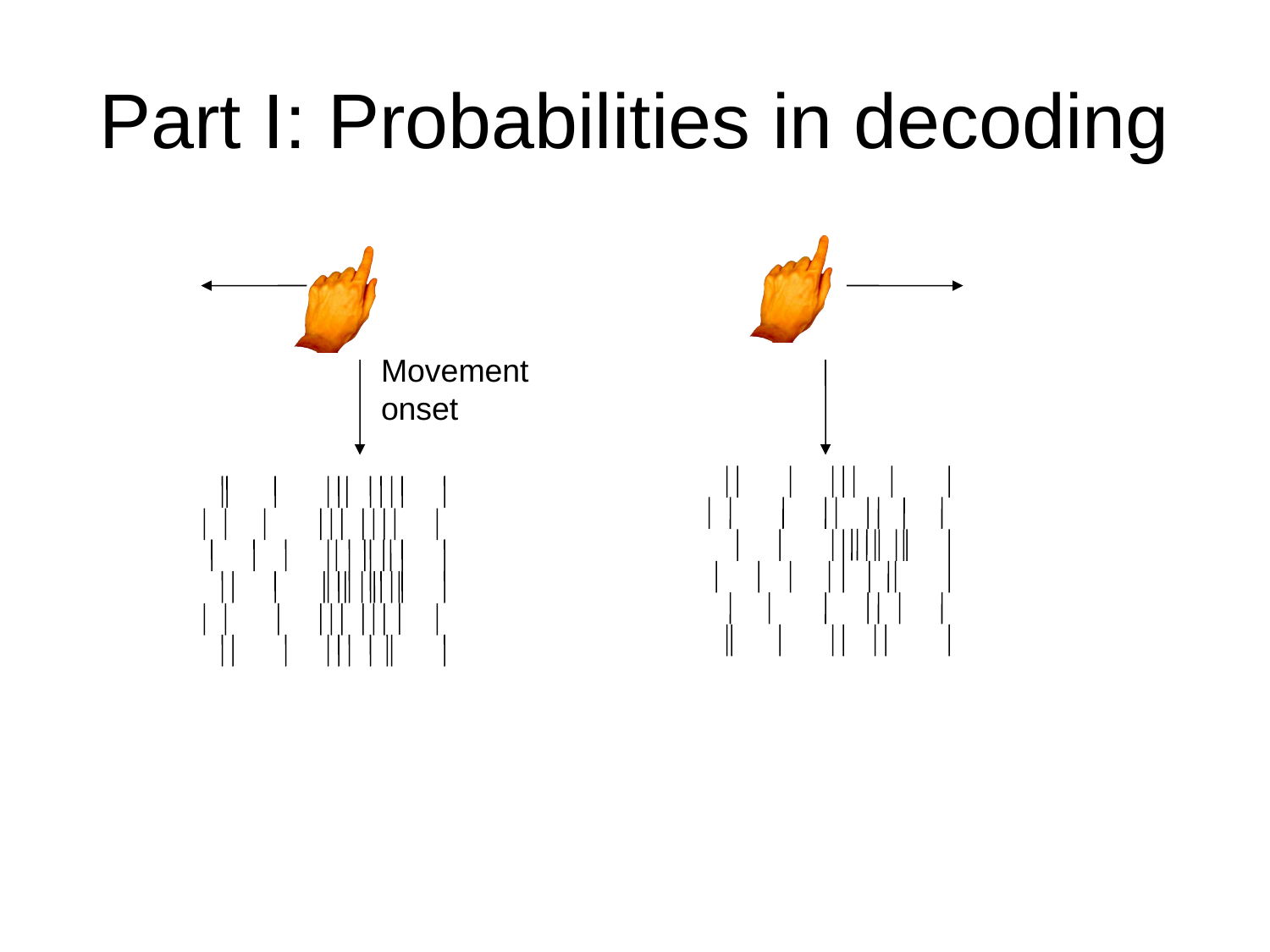

# Part I: Probabilities in decoding
Movement
onset

## Slide 9
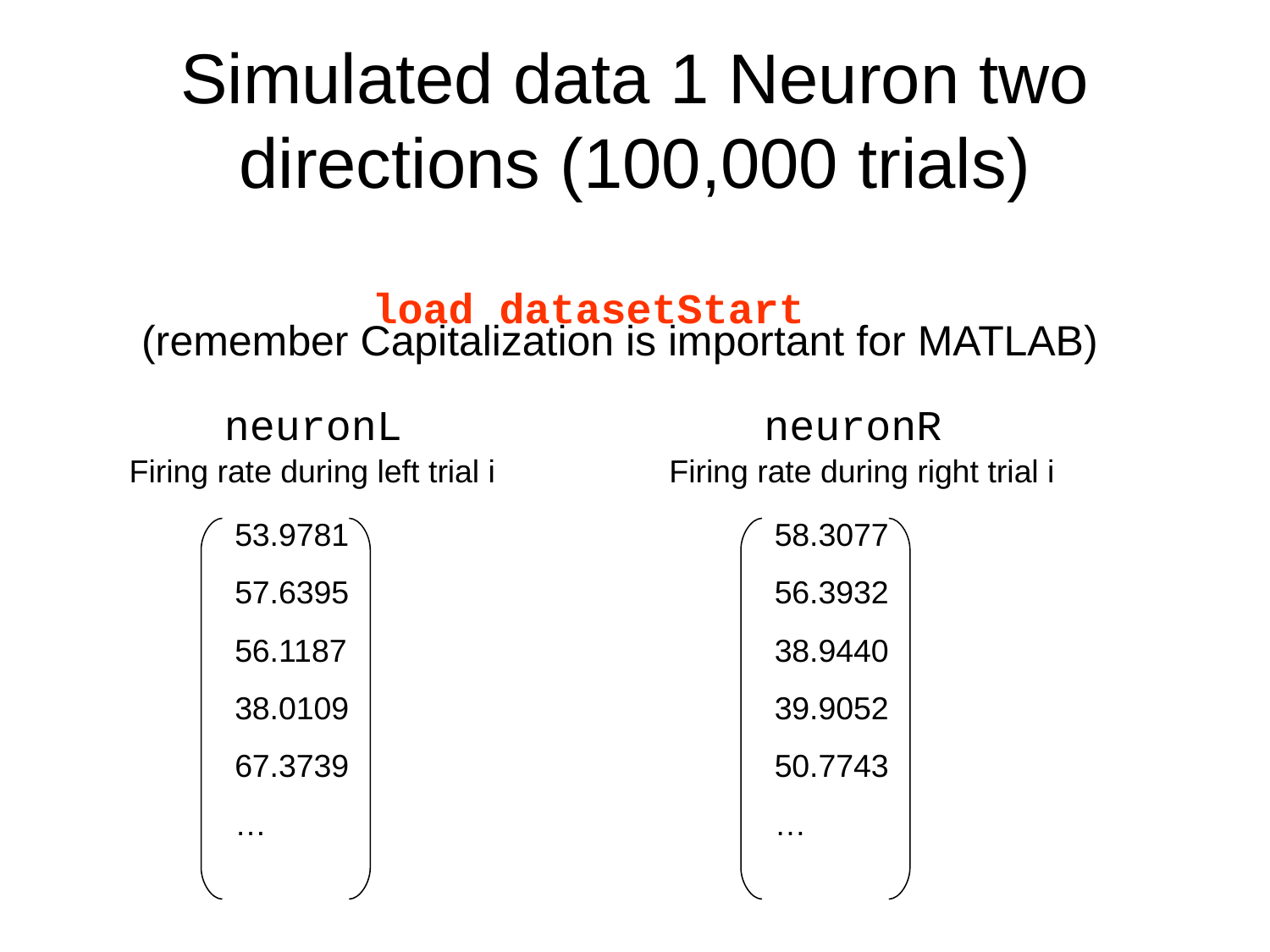

# Simulated data 1 Neuron two directions (100,000 trials)
load datasetStart
(remember Capitalization is important for MATLAB)
neuronL
neuronR
Firing rate during left trial i
Firing rate during right trial i
53.9781
57.6395
56.1187
38.0109
67.3739
…
58.3077
56.3932
38.9440
39.9052
50.7743
…

## Slide 10
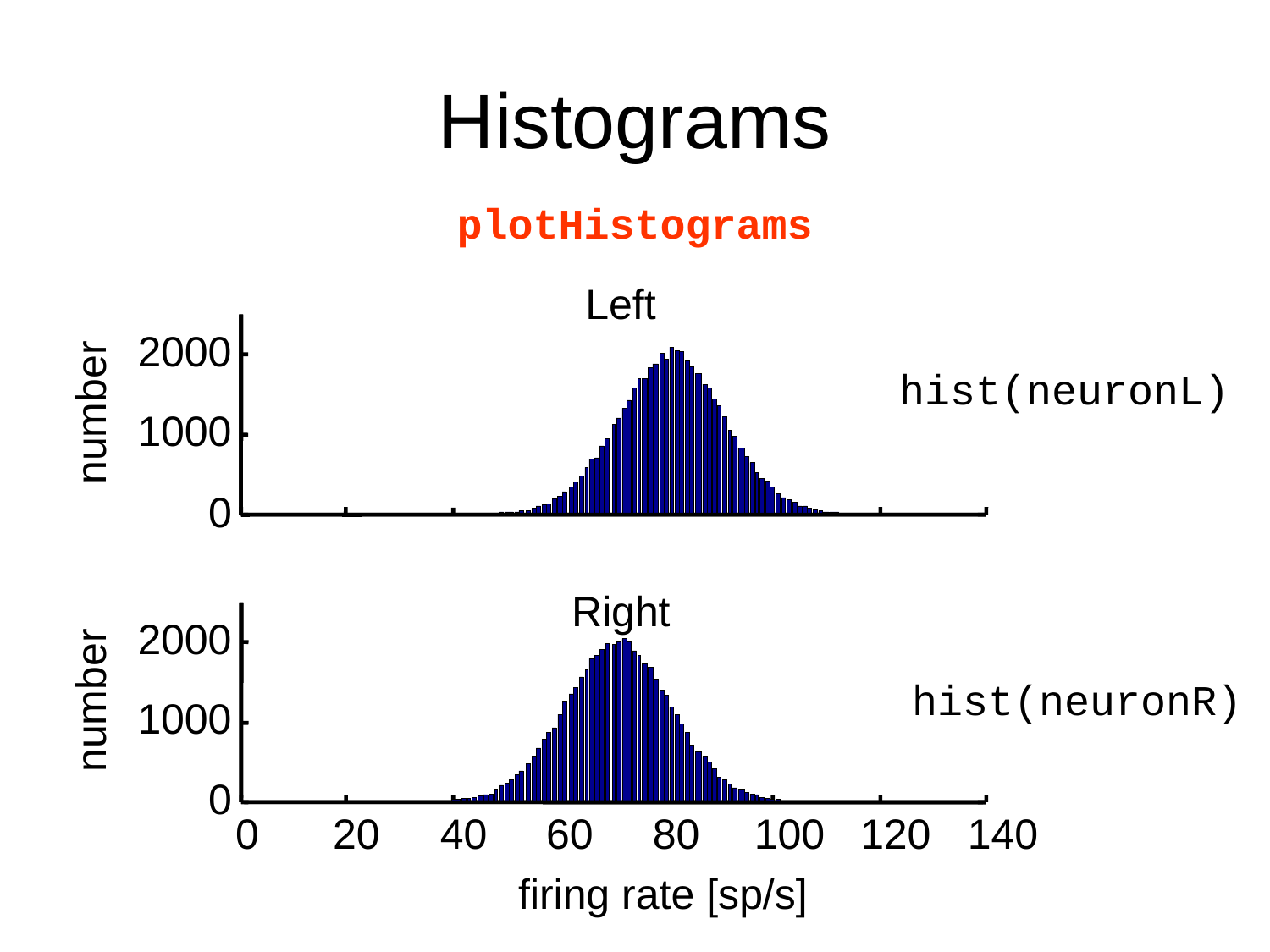

# Histograms
plotHistograms
Left
2000
hist(neuronL)
number
1000
0
Right
2000
hist(neuronR)
number
1000
0
0
20
40
60
80
100
120
140
firing rate [sp/s]

## Slide 11
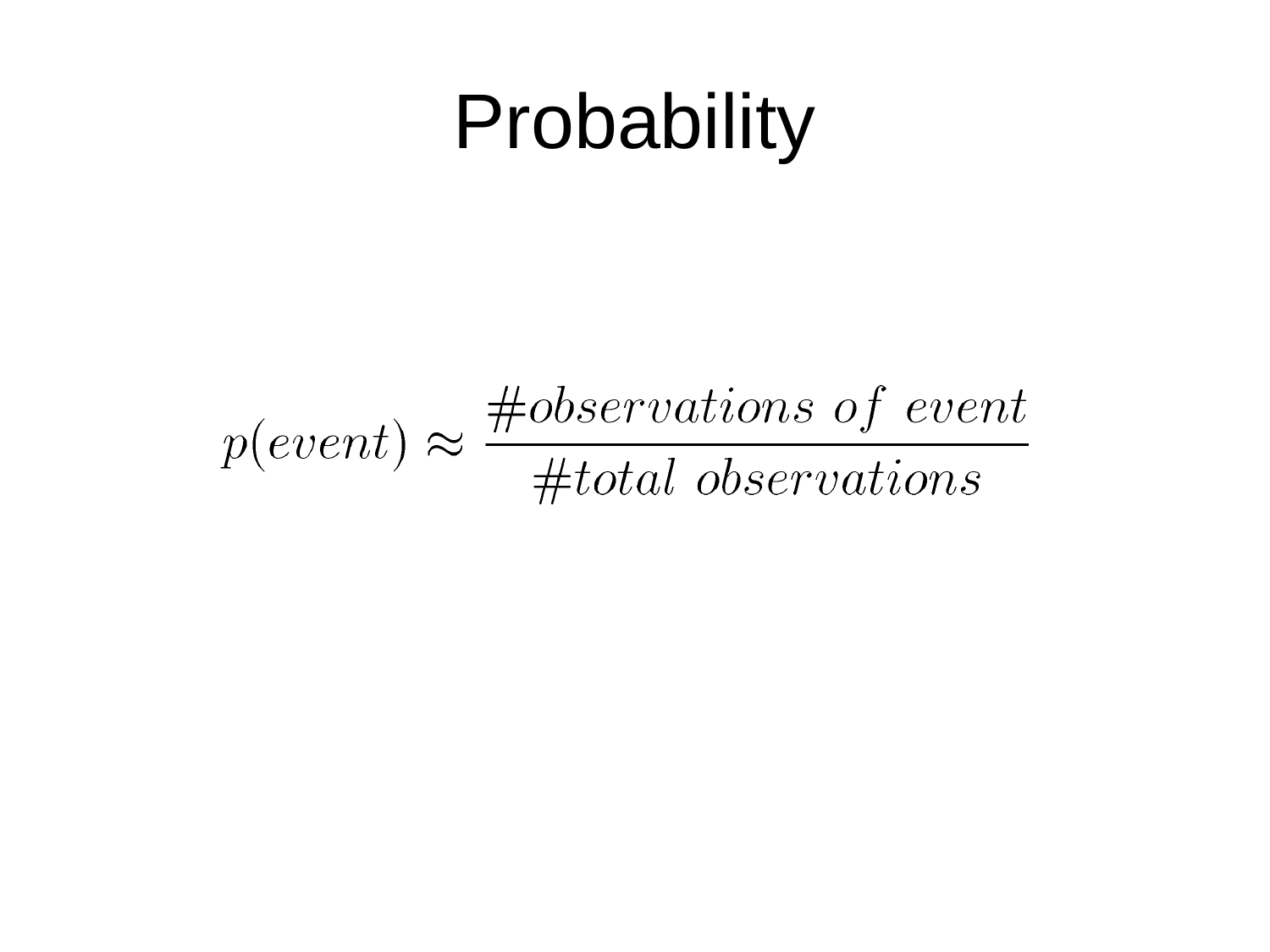

# Probability

## Slide 12
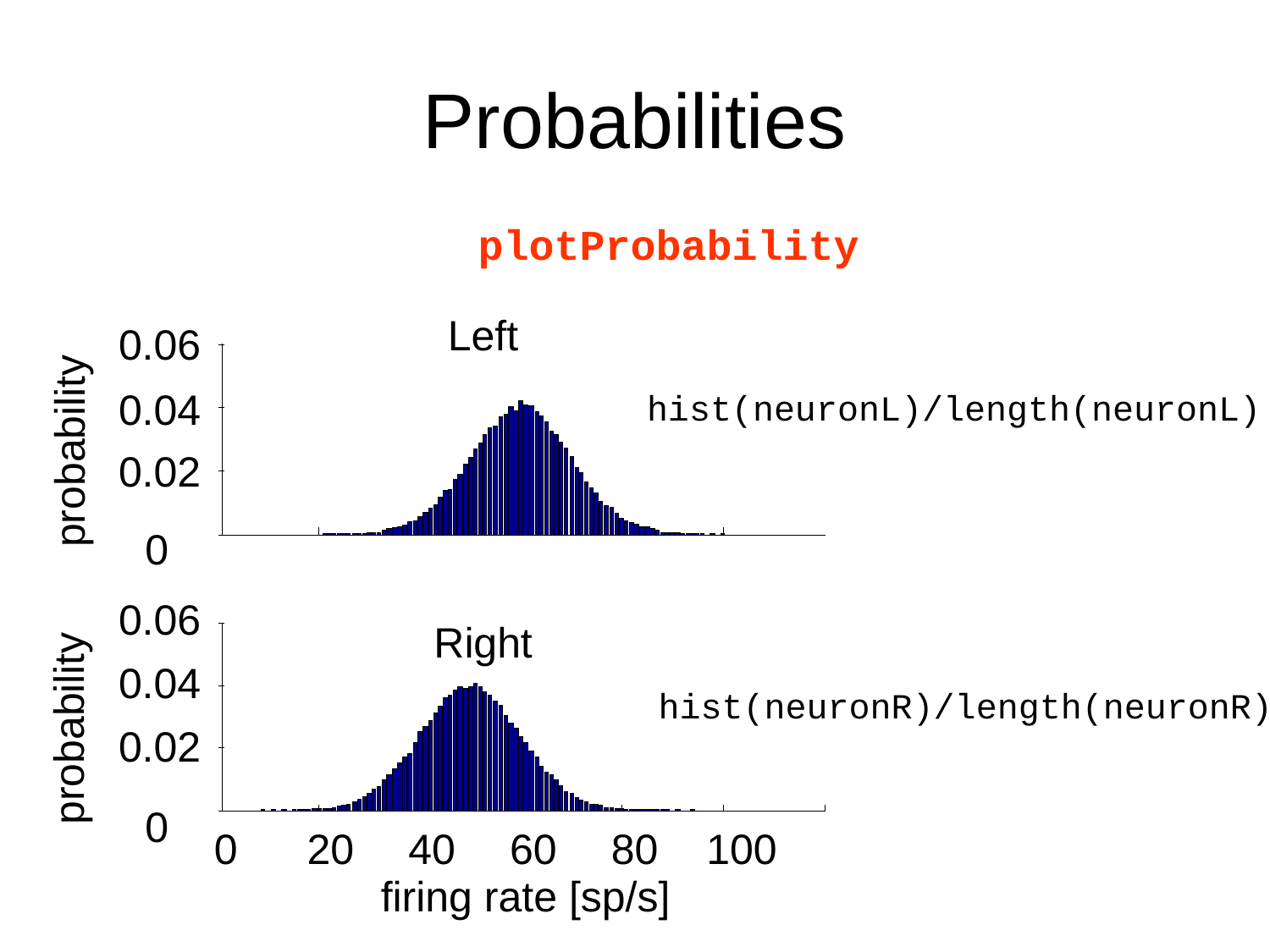

# Probabilities
plotProbability
Left
0.06
hist(neuronL)/length(neuronL)
0.04
probability
0.02
0
0.06
Right
0.04
hist(neuronR)/length(neuronR)
probability
0.02
0
0
20
40
60
80
100
firing rate [sp/s]

## Slide 13
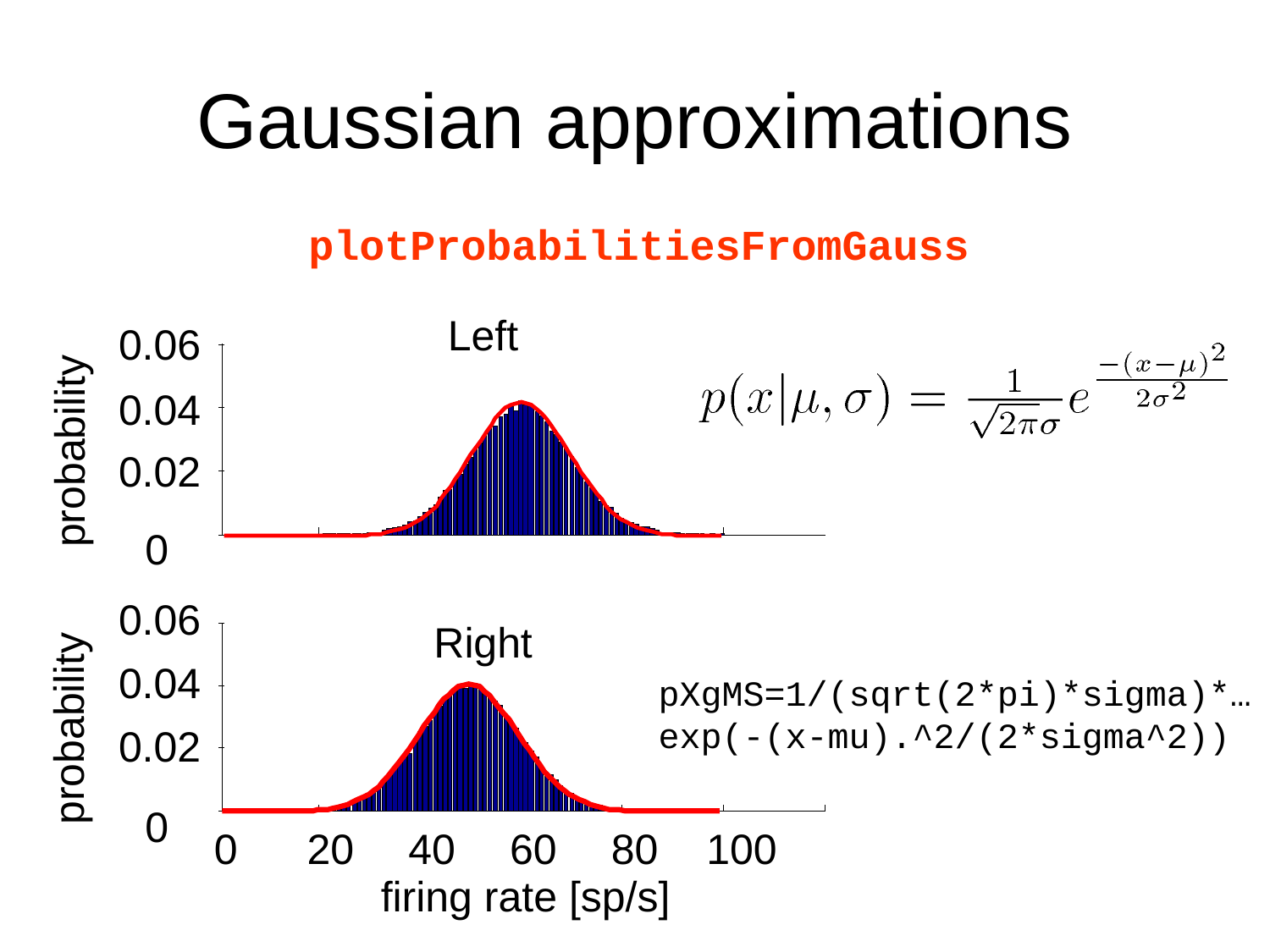

# Gaussian approximations
plotProbabilitiesFromGauss
Left
0.06
0.04
probability
0.02
0
0.06
Right
0.04
pXgMS=1/(sqrt(2*pi)*sigma)*…
exp(-(x-mu).^2/(2*sigma^2))
probability
0.02
0
0
20
40
60
80
100
firing rate [sp/s]

## Slide 14
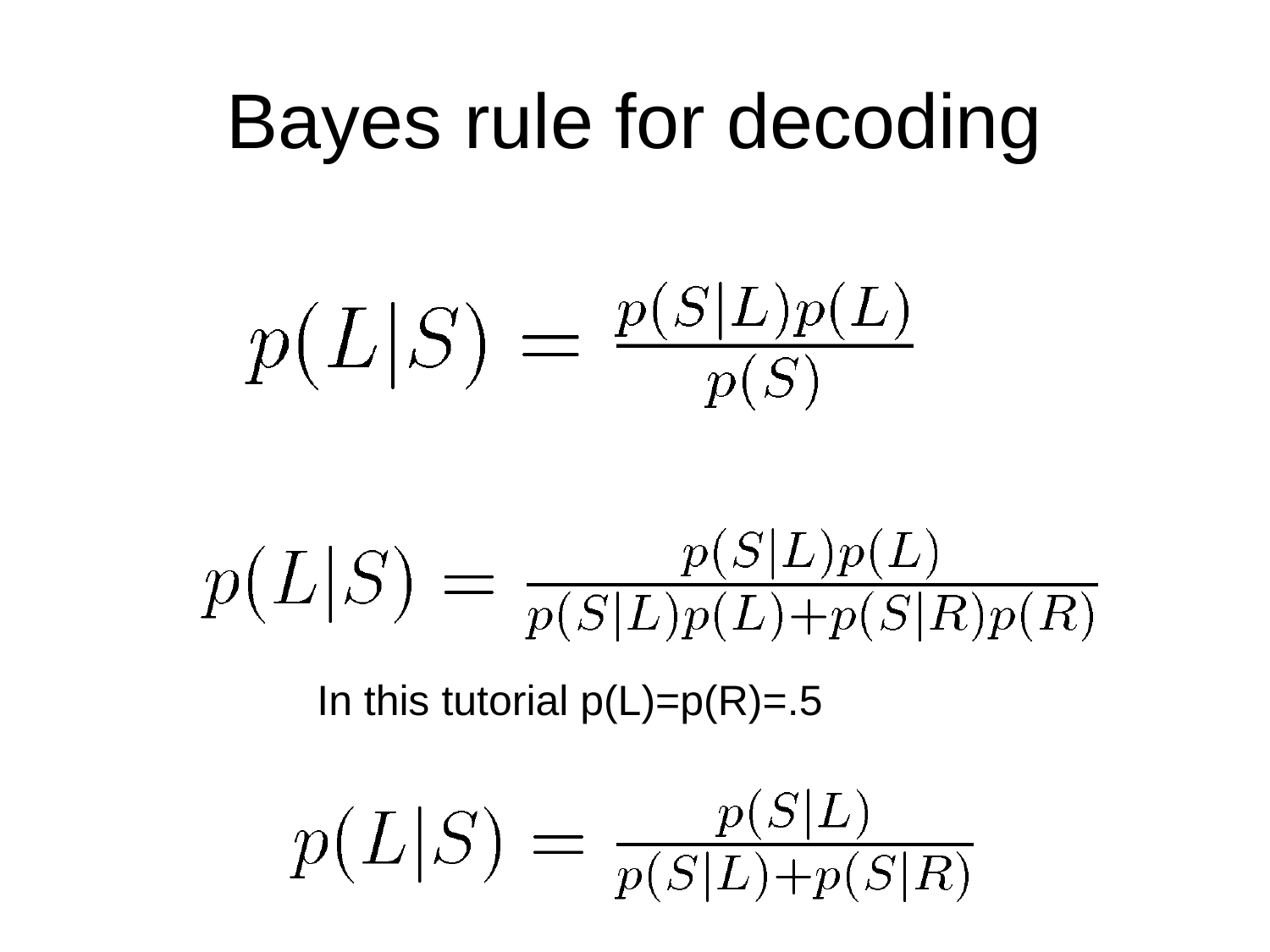

# Bayes rule for decoding
In this tutorial p(L)=p(R)=.5

## Slide 15
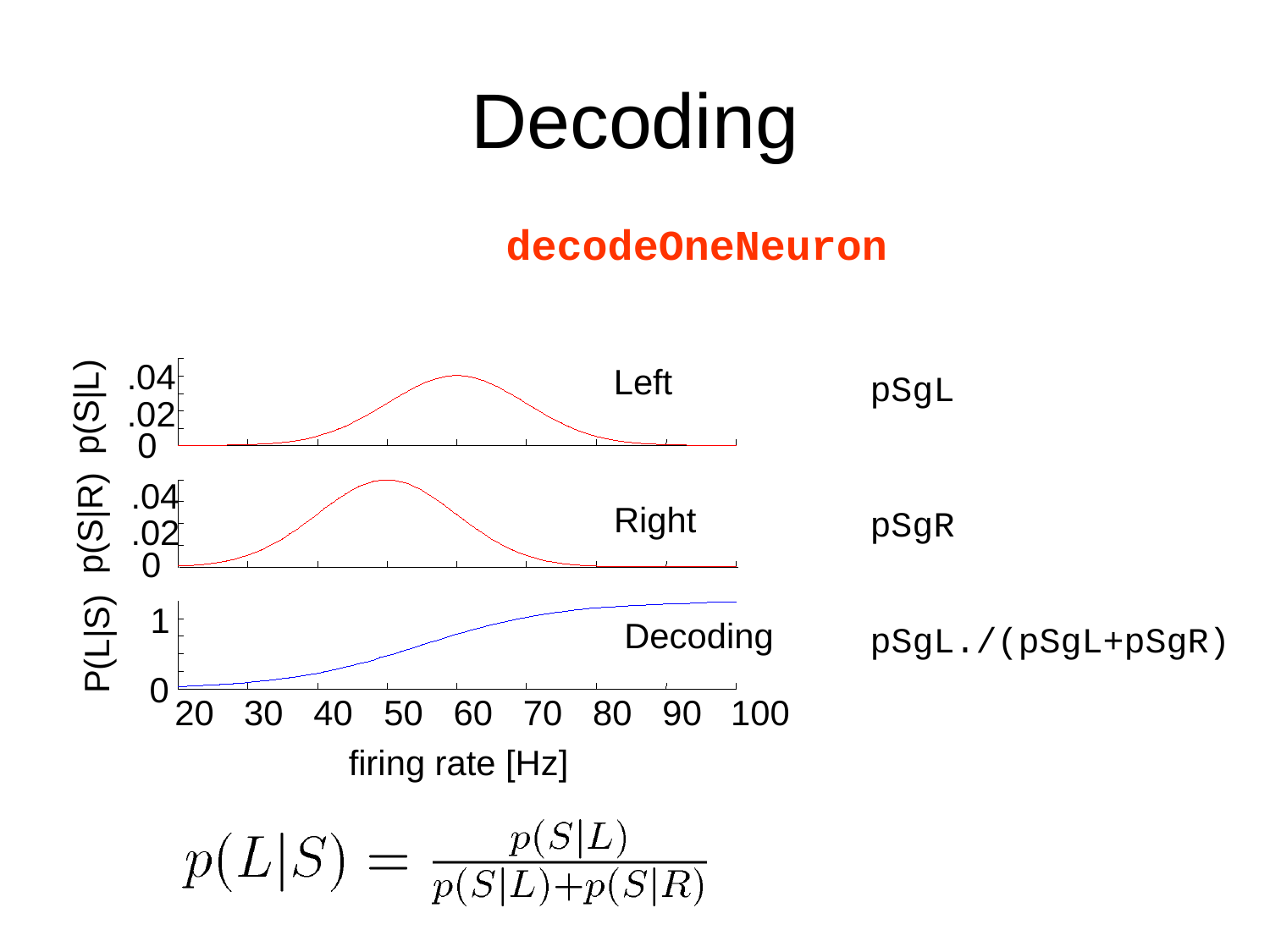

# Decoding
decodeOneNeuron
.04
Left
pSgL
p(S|L)
.02
0
.04
pSgR
Right
p(S|R)
.02
0
1
pSgL./(pSgL+pSgR)
Decoding
P(L|S)
0
20
30
40
50
60
70
80
90
100
firing rate [Hz]

## Slide 16
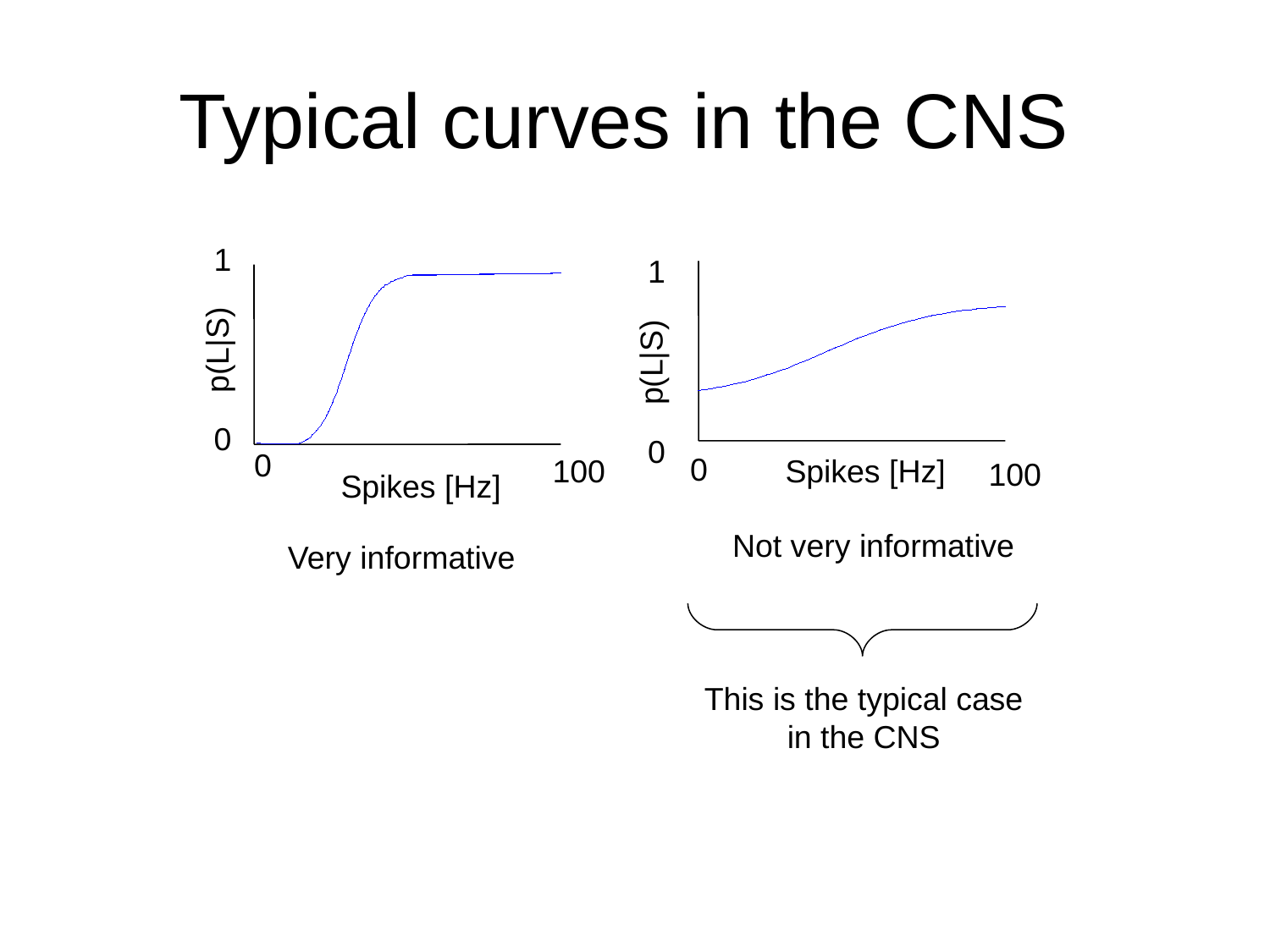

# Typical curves in the CNS
1
1
p(L|S)
p(L|S)
0
0
0
0
100
Spikes [Hz]
100
Spikes [Hz]
Not very informative
Very informative
This is the typical case
in the CNS

## Slide 17
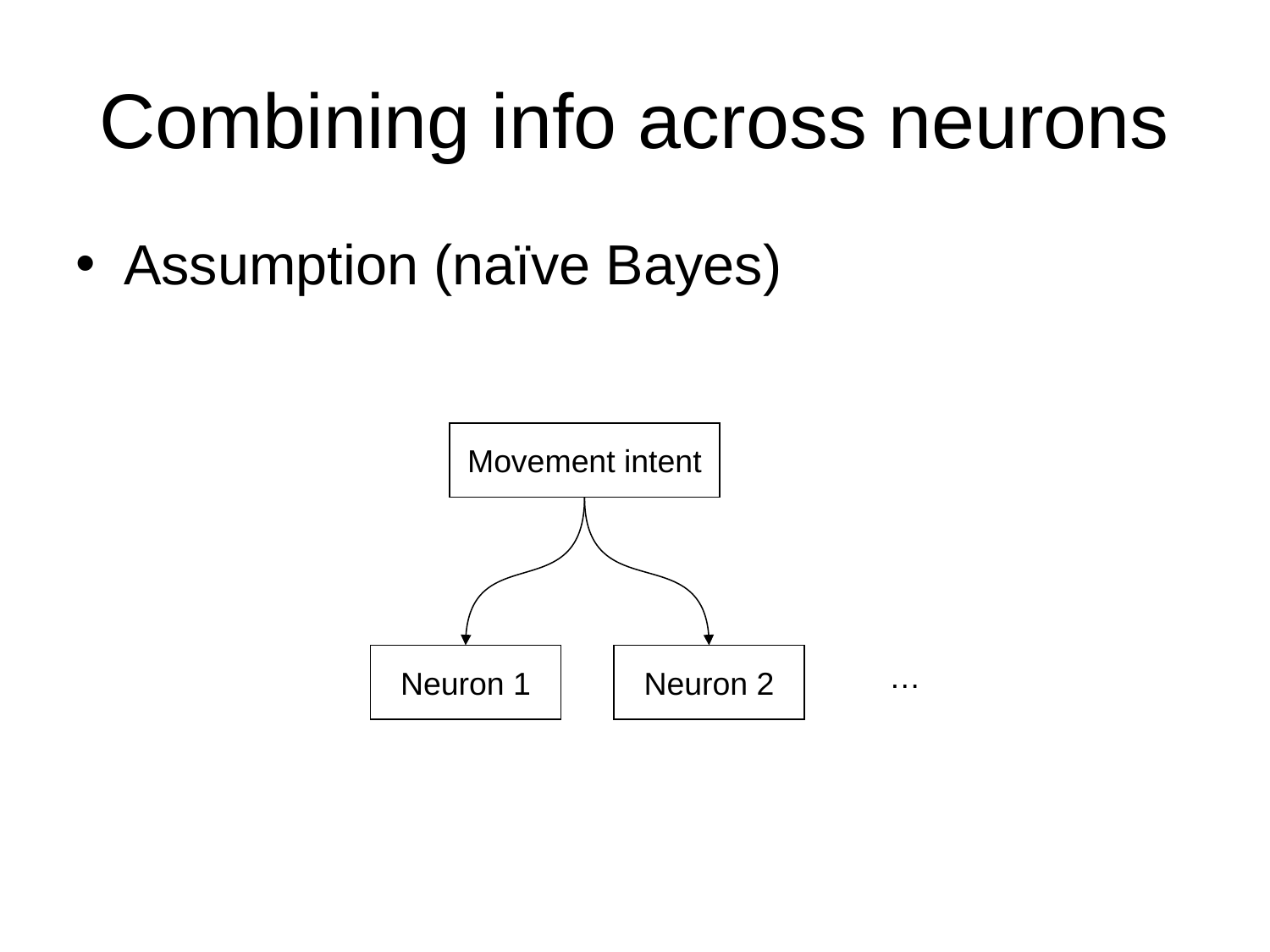

# Combining info across neurons
Assumption (naïve Bayes)
Movement intent
Neuron 1
Neuron 2
…

## Slide 18
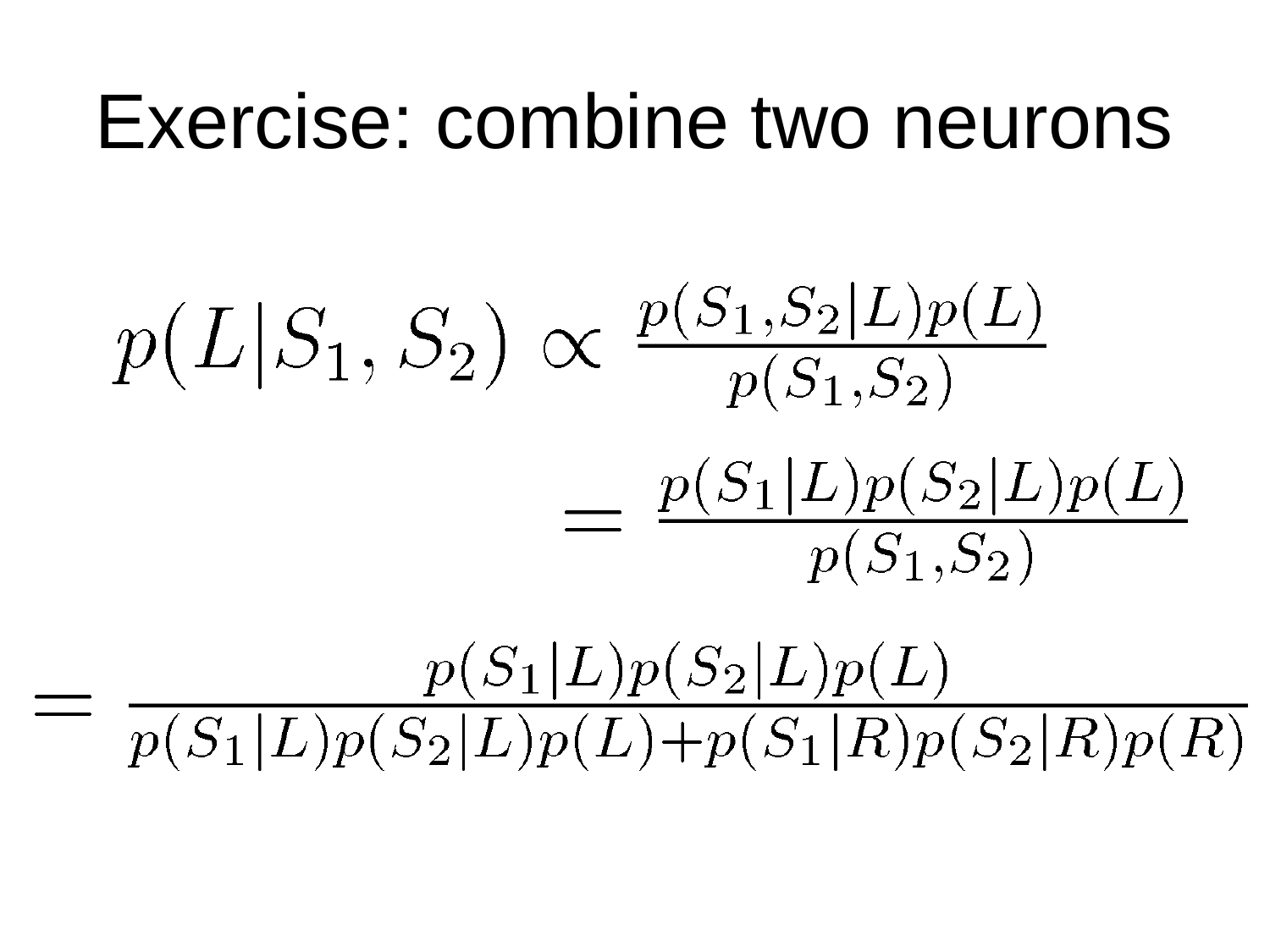

# Exercise: combine two neurons

## Slide 19
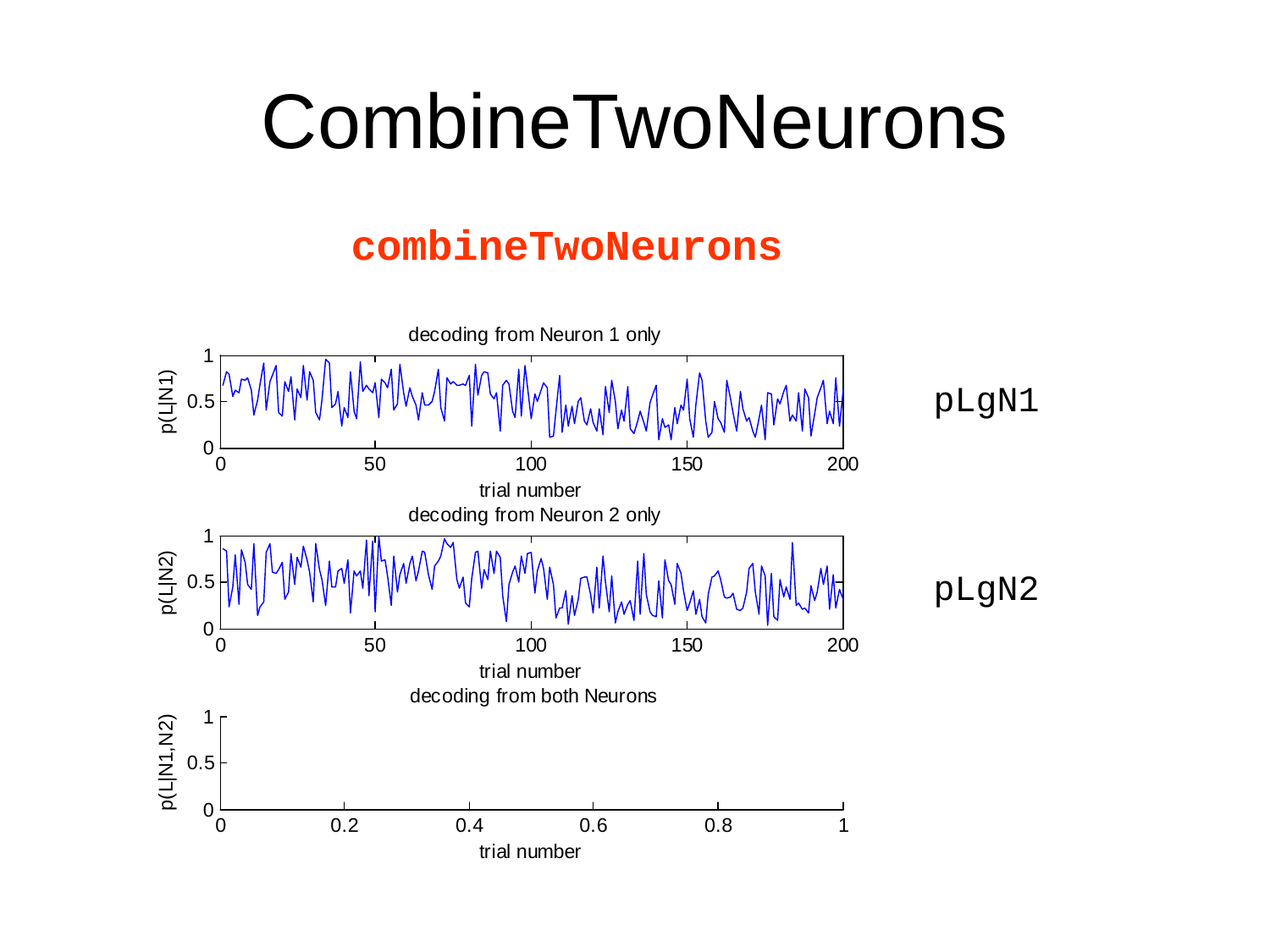

# CombineTwoNeurons
combineTwoNeurons
pLgN1
pLgN2

## Slide 20
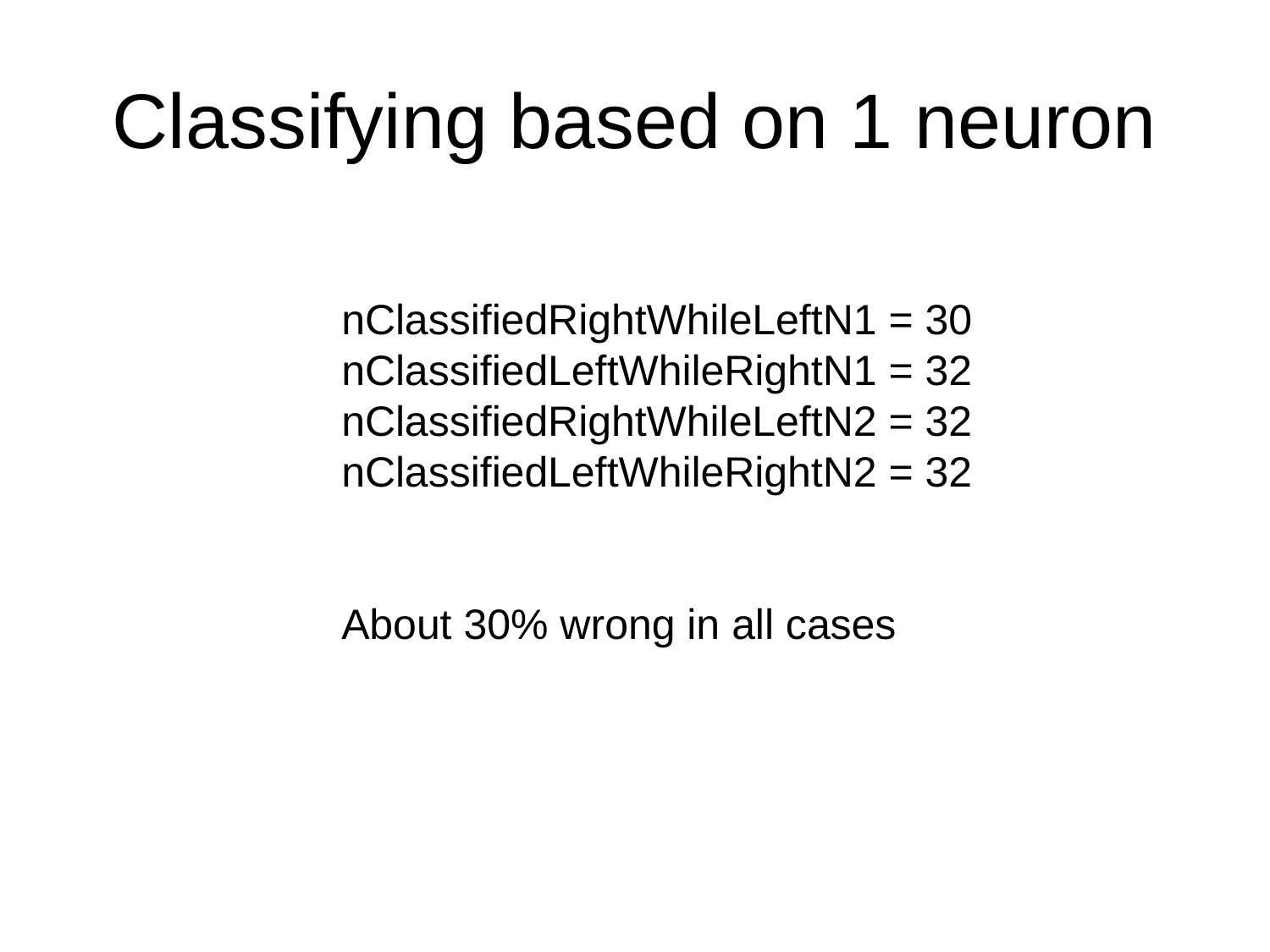

# Classifying based on 1 neuron
nClassifiedRightWhileLeftN1 = 30
nClassifiedLeftWhileRightN1 = 32
nClassifiedRightWhileLeftN2 = 32
nClassifiedLeftWhileRightN2 = 32
About 30% wrong in all cases

## Slide 21
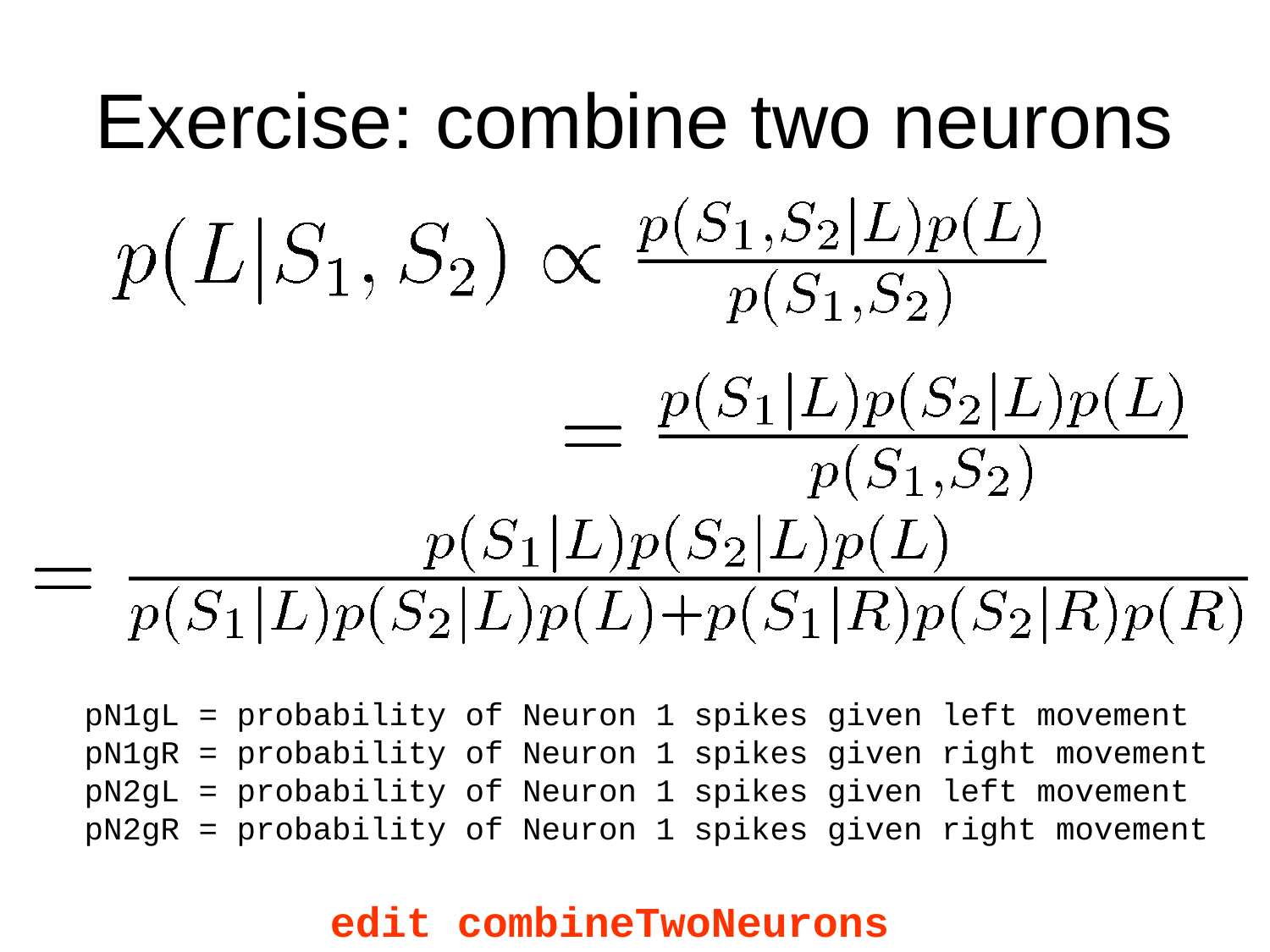

# Exercise: combine two neurons
pN1gL = probability of Neuron 1 spikes given left movement
pN1gR = probability of Neuron 1 spikes given right movement
pN2gL = probability of Neuron 1 spikes given left movement
pN2gR = probability of Neuron 1 spikes given right movement
edit combineTwoNeurons

## Slide 22
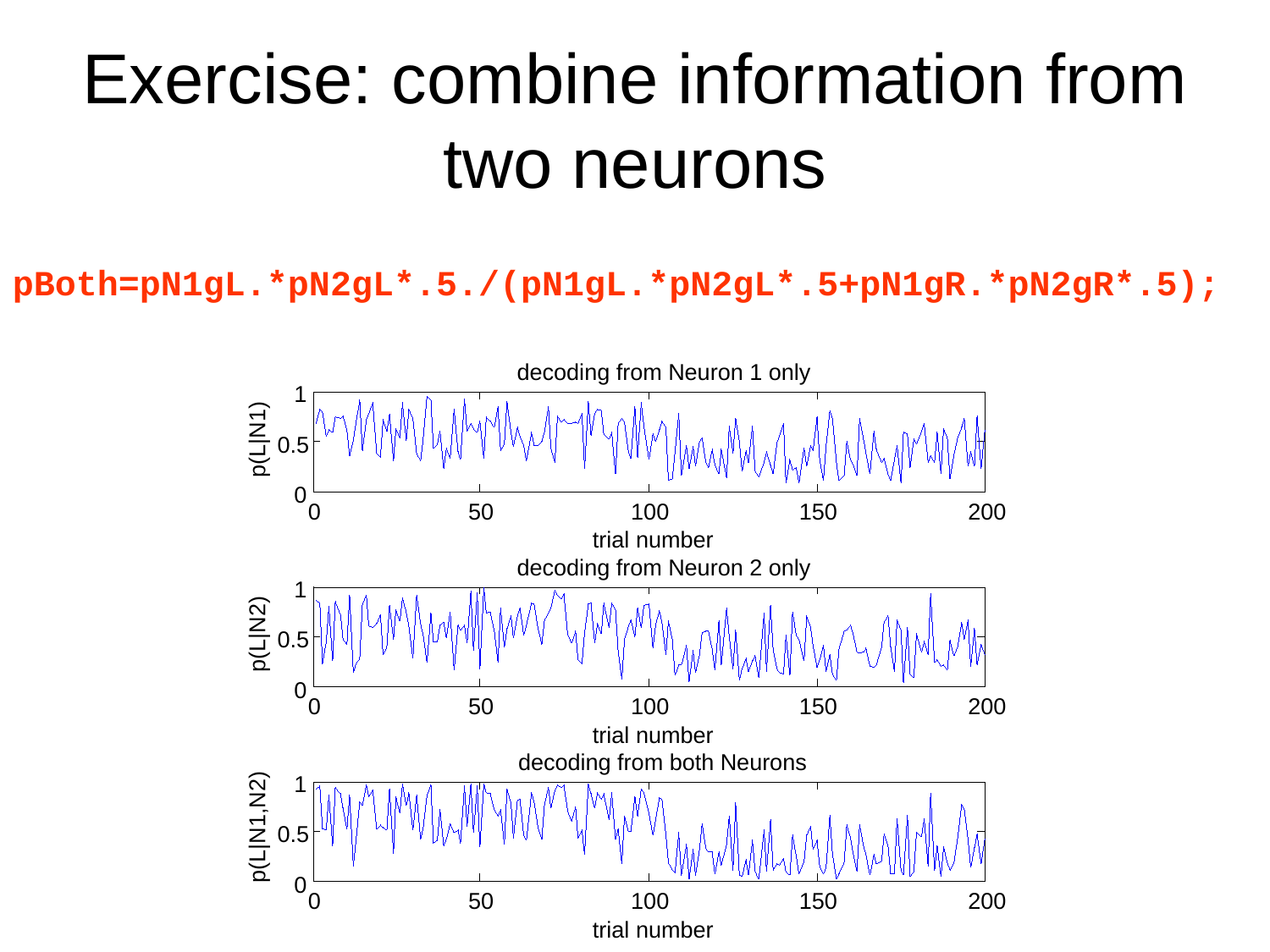

# Exercise: combine information from two neurons
pBoth=pN1gL.*pN2gL*.5./(pN1gL.*pN2gL*.5+pN1gR.*pN2gR*.5);
decoding from Neuron 1 only
1
p(L|N1)
0.5
0
0
50
100
150
200
trial number
decoding from Neuron 2 only
1
p(L|N2)
0.5
0
0
50
100
150
200
trial number
decoding from both Neurons
1
p(L|N1,N2)
0.5
0
0
50
100
150
200
trial number

## Slide 23
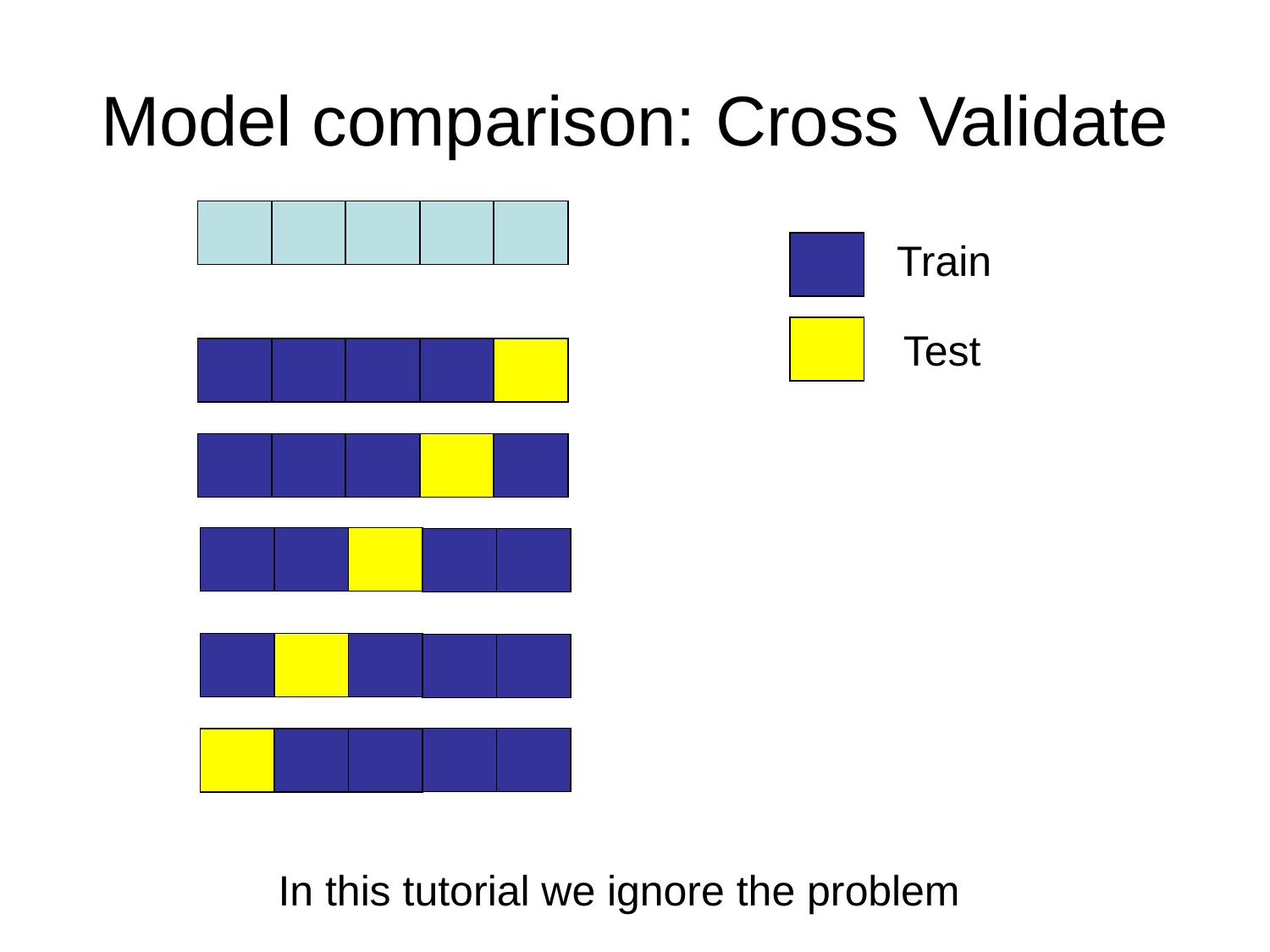

# Model comparison: Cross Validate
Train
Test
In this tutorial we ignore the problem

## Slide 24
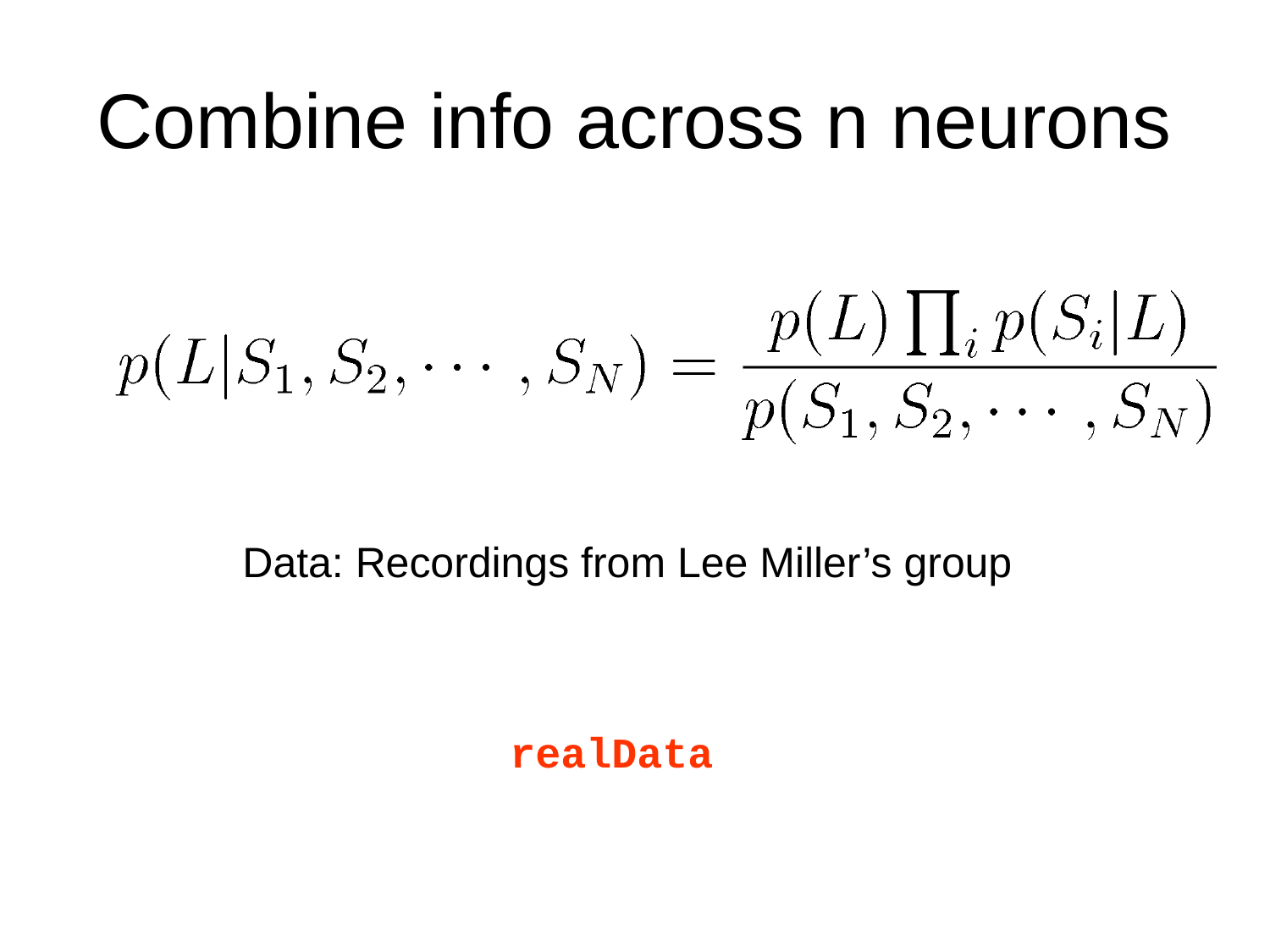

# Combine info across n neurons
Data: Recordings from Lee Miller’s group
realData

## Slide 25
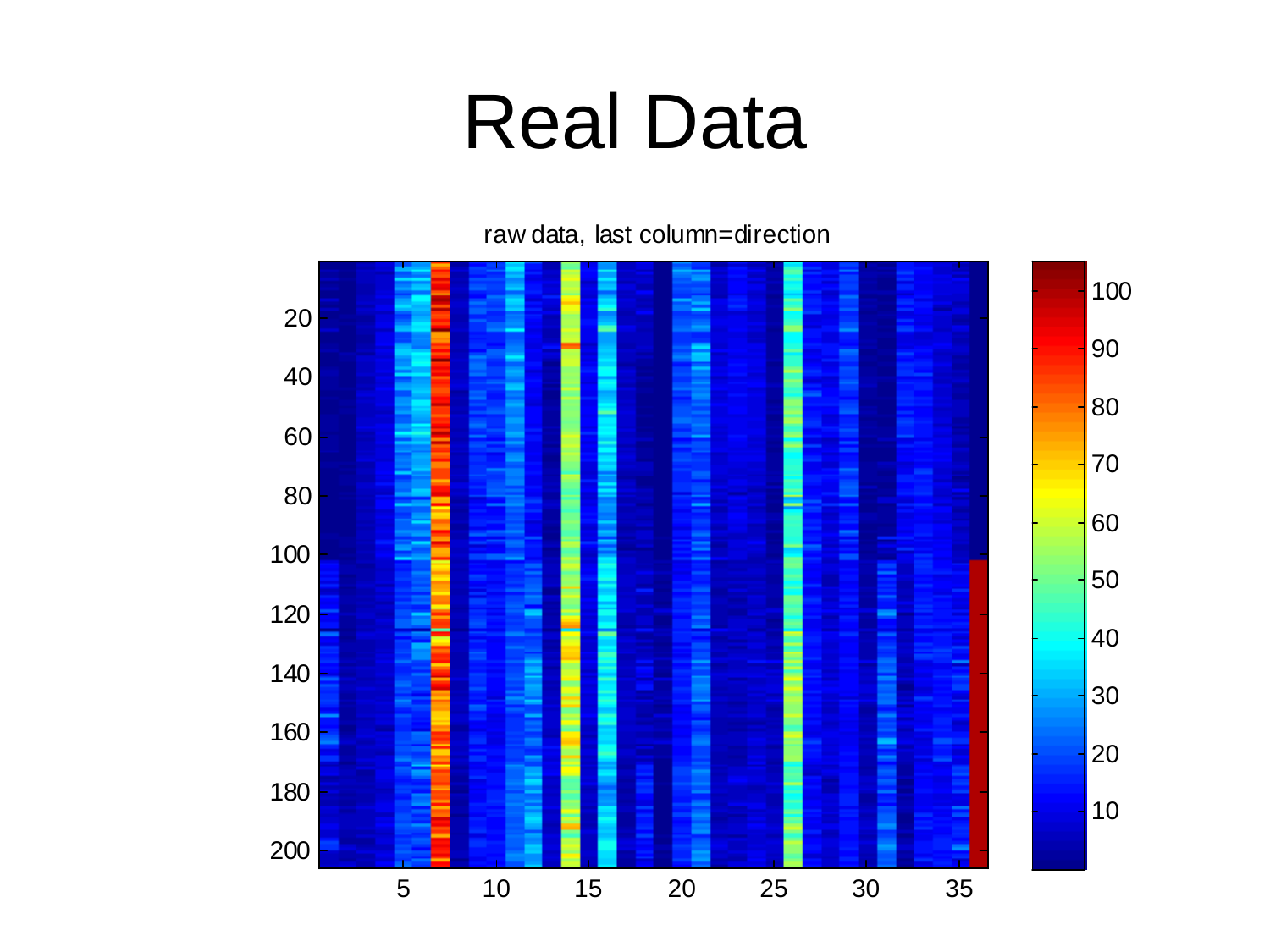

# Real Data

## Slide 26
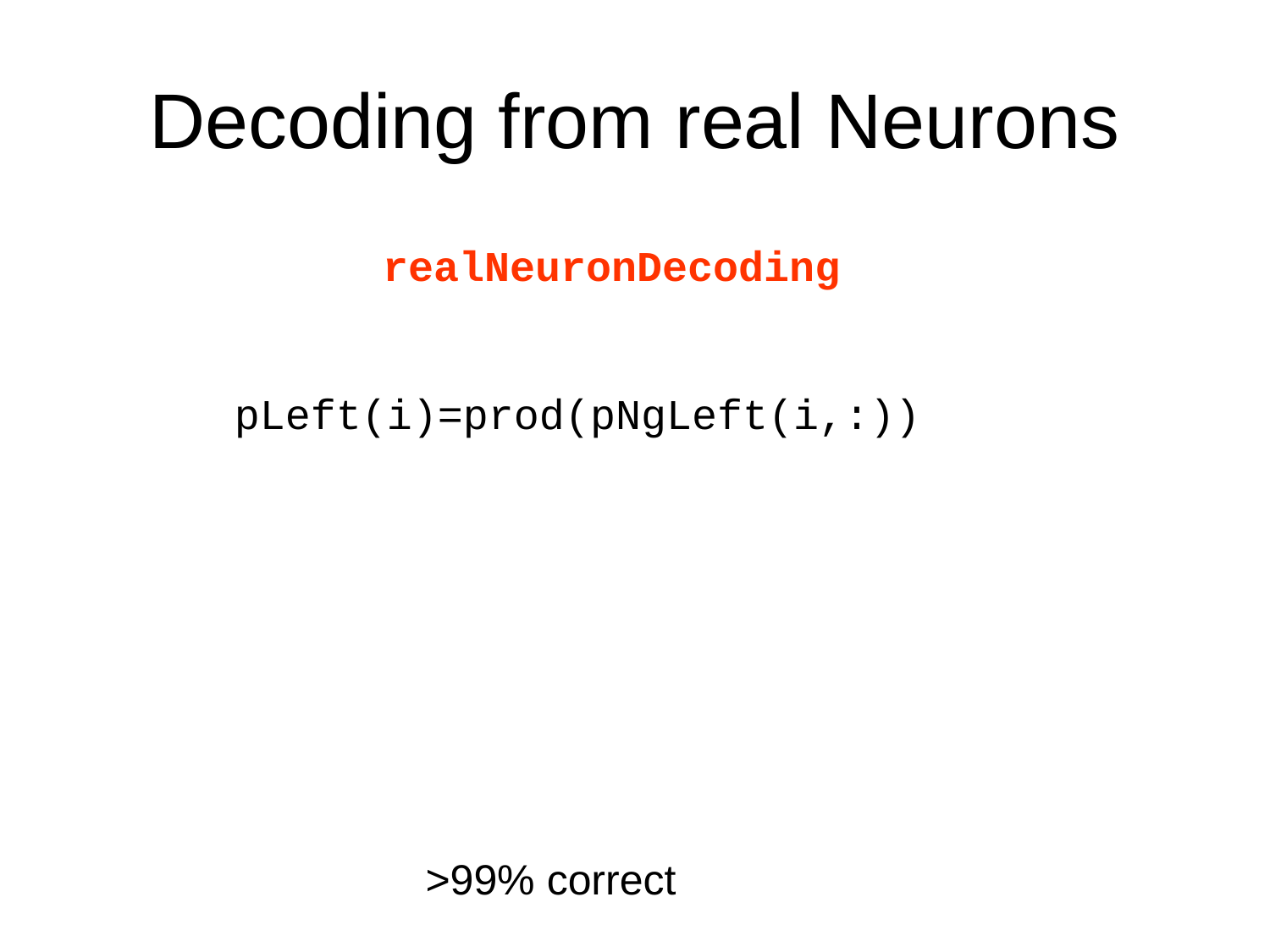

# Decoding from real Neurons
realNeuronDecoding
pLeft(i)=prod(pNgLeft(i,:))
>99% correct

## Slide 27
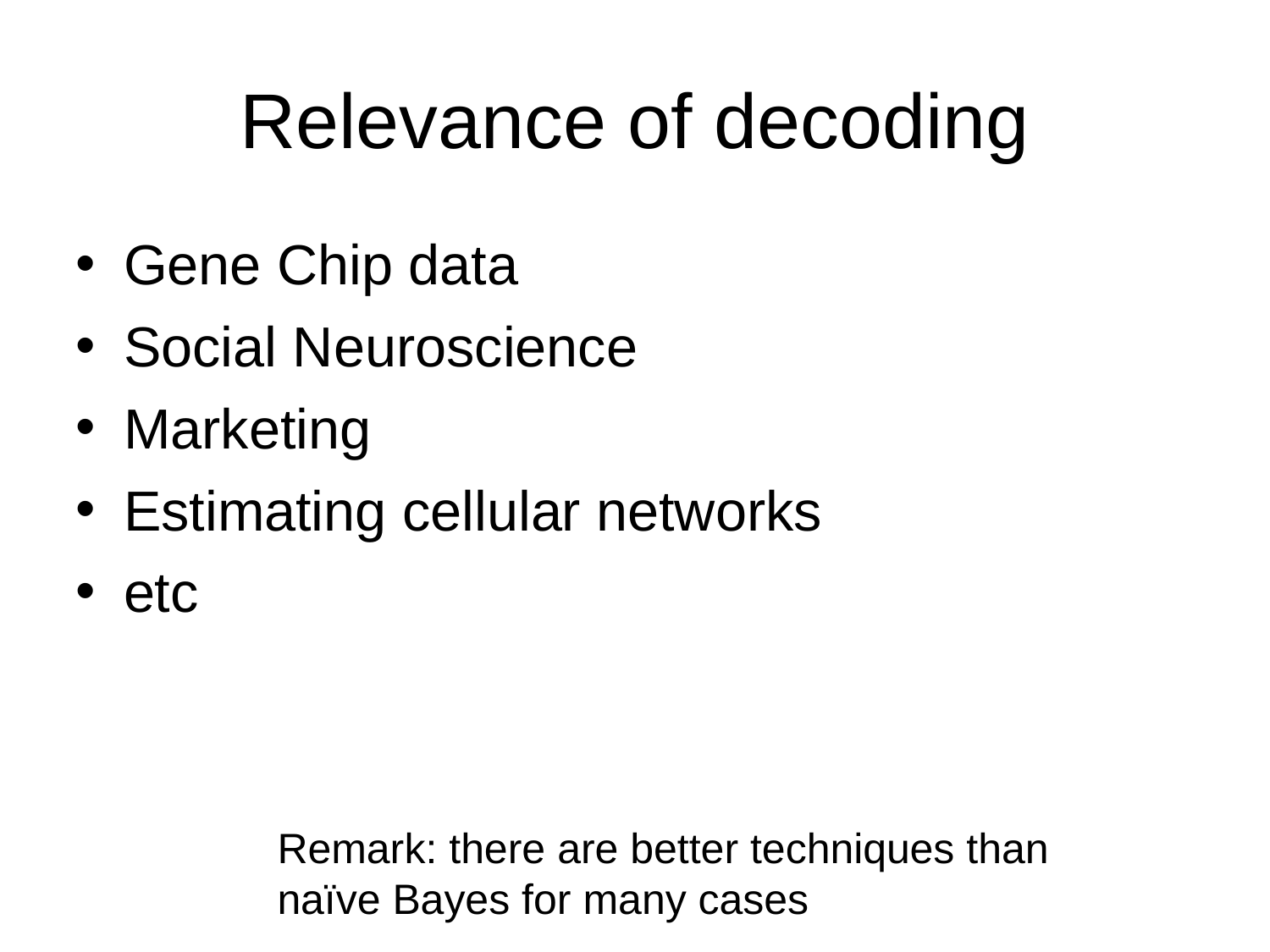

# Relevance of decoding
Gene Chip data
Social Neuroscience
Marketing
Estimating cellular networks
etc
Remark: there are better techniques than
naïve Bayes for many cases

## Slide 28
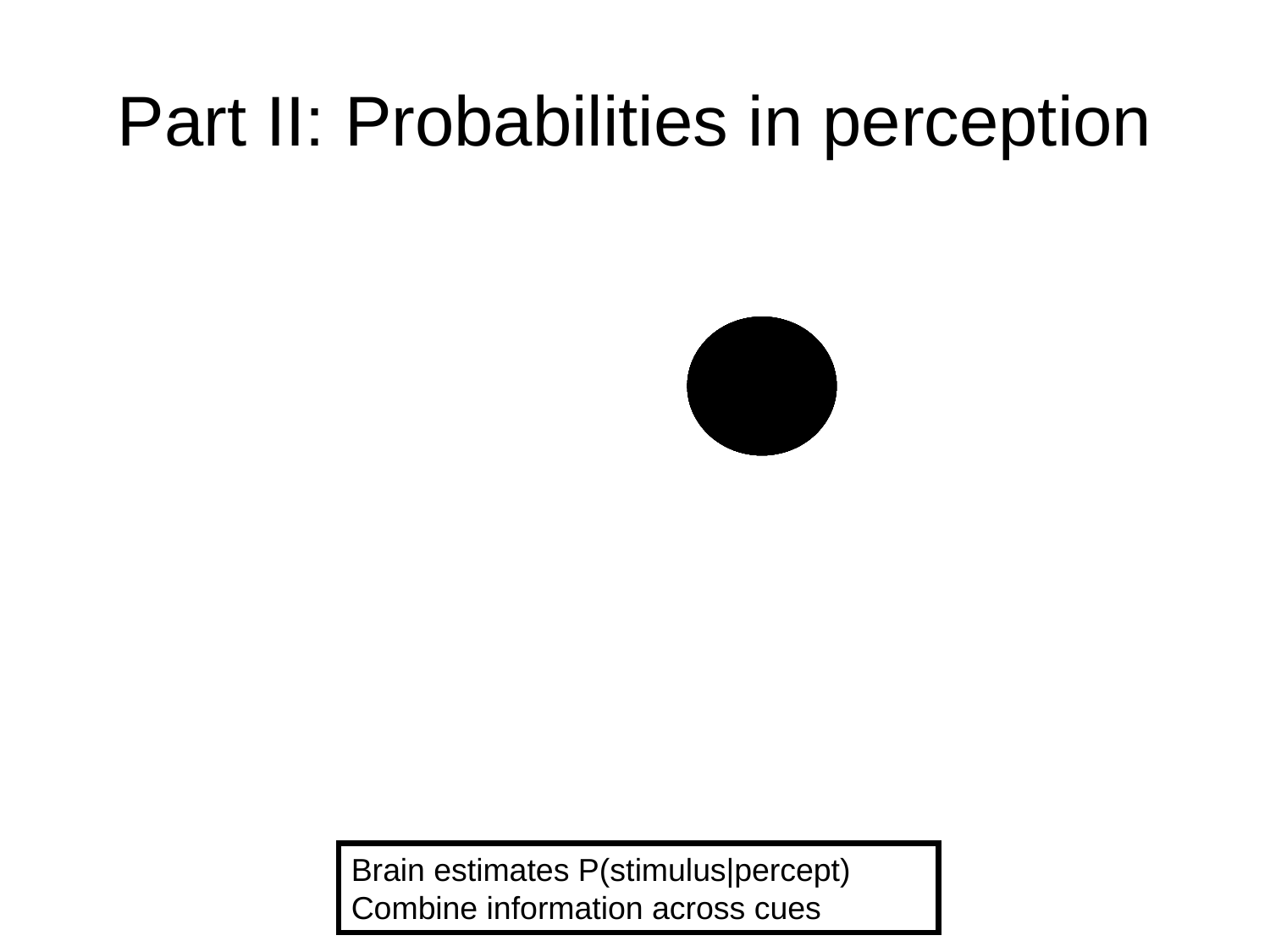

# Part II: Probabilities in perception
Brain estimates P(stimulus|percept)
Combine information across cues

## Slide 29
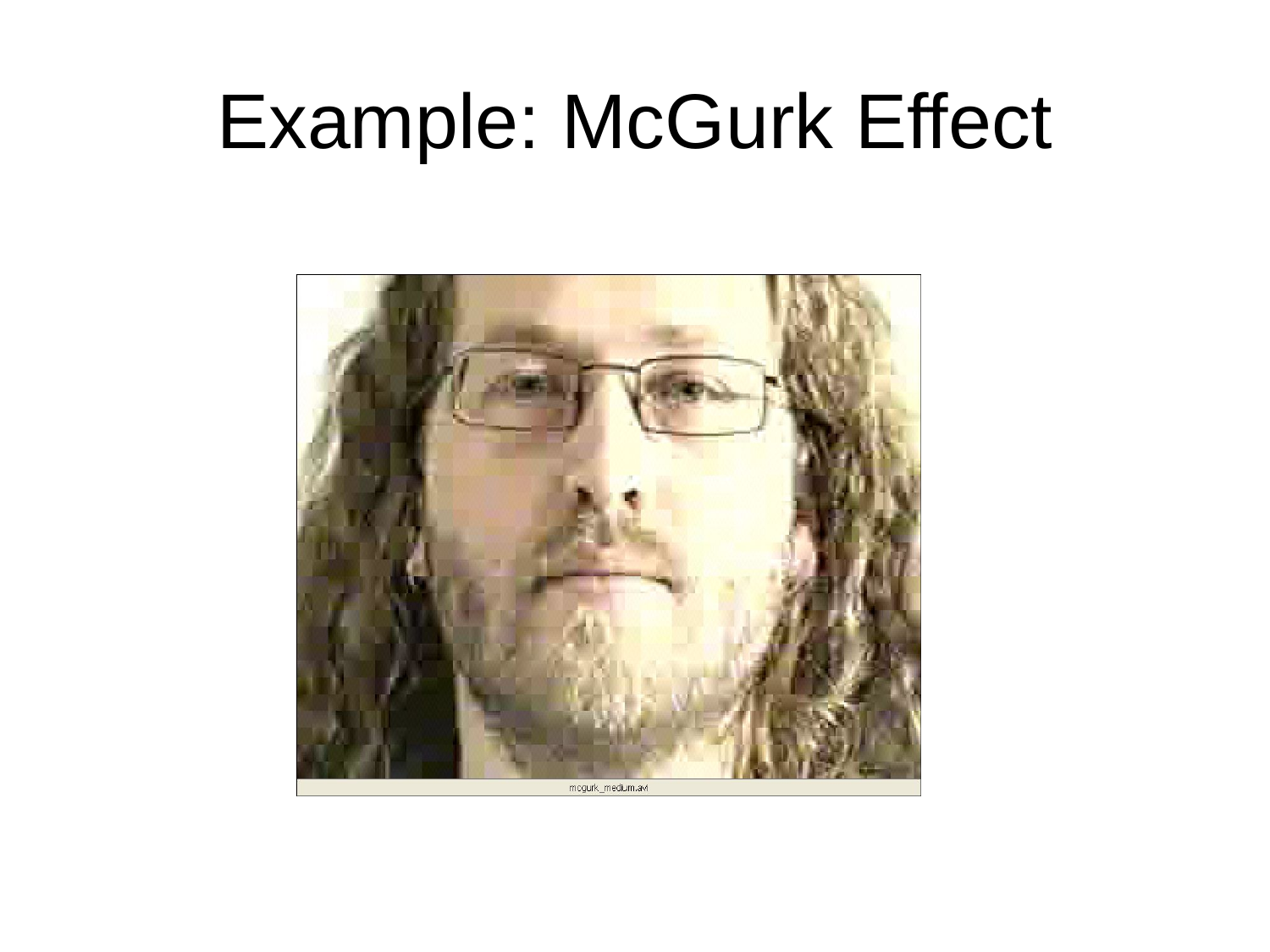

# Example: McGurk Effect

## Slide 30
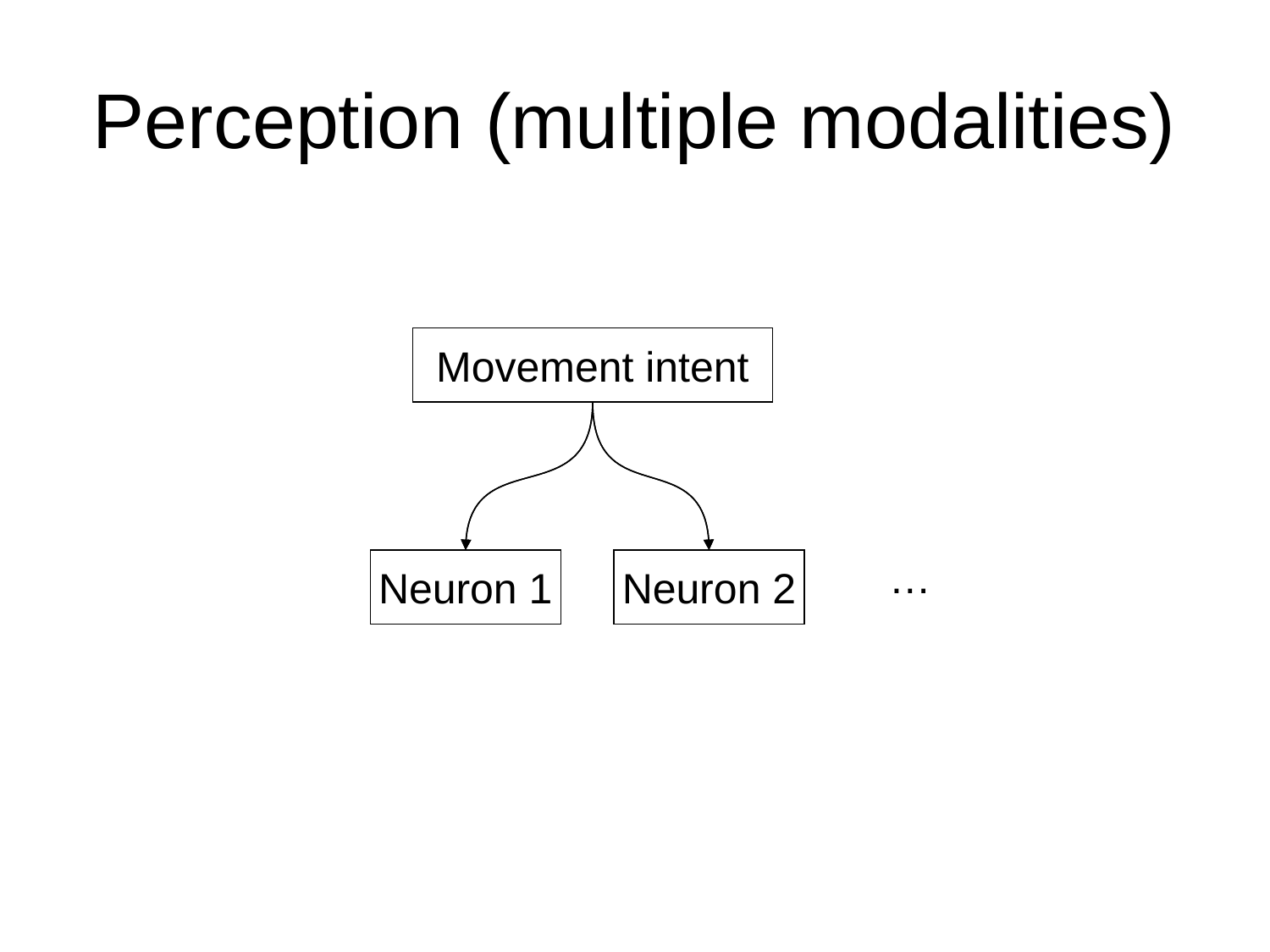

# Perception (multiple modalities)
World state
Movement intent
…
…
Sensor 1
Neuron 1
Sensor 2
Neuron 2

## Slide 31
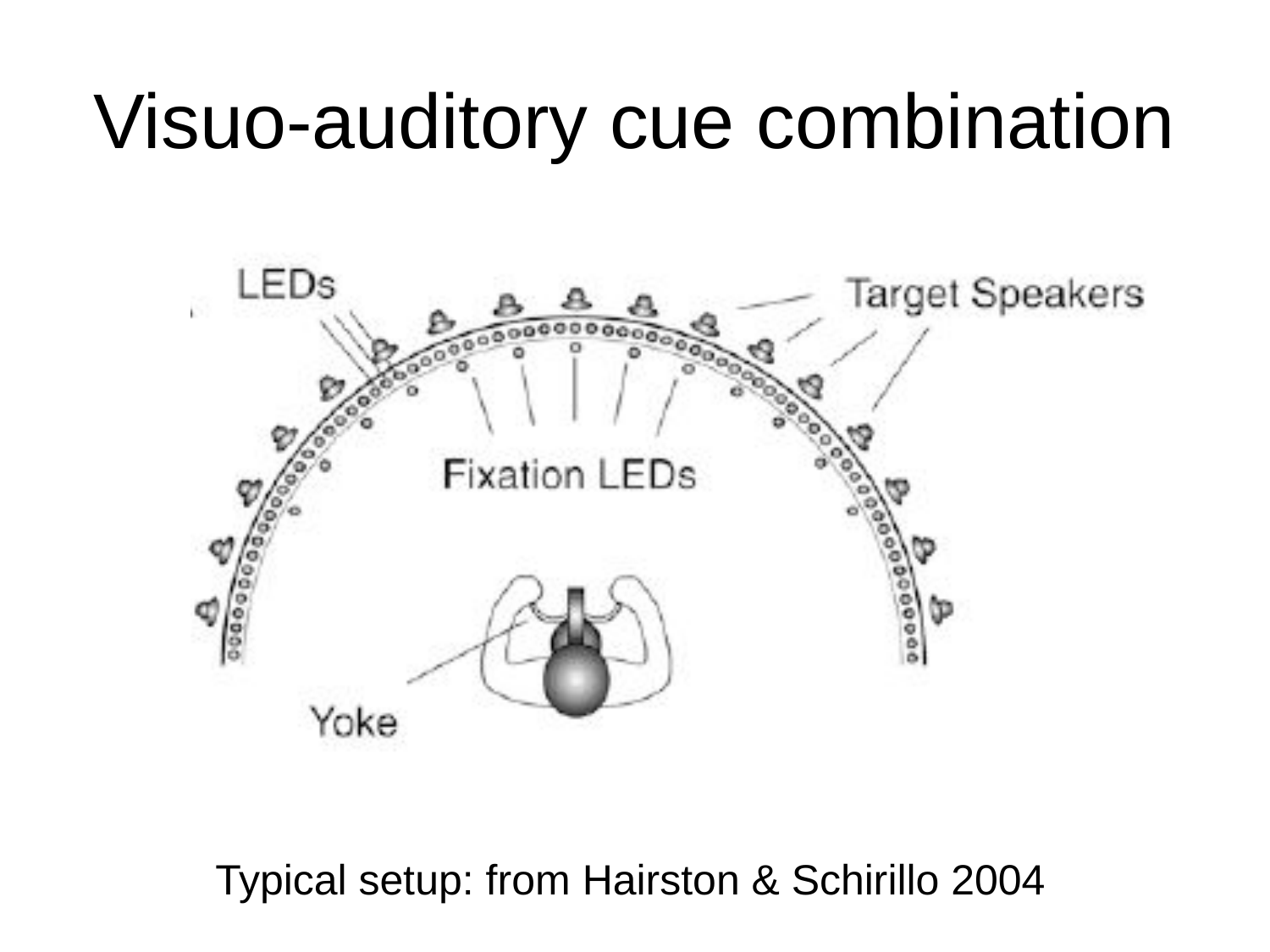

# Visuo-auditory cue combination
Typical setup: from Hairston & Schirillo 2004

## Slide 32
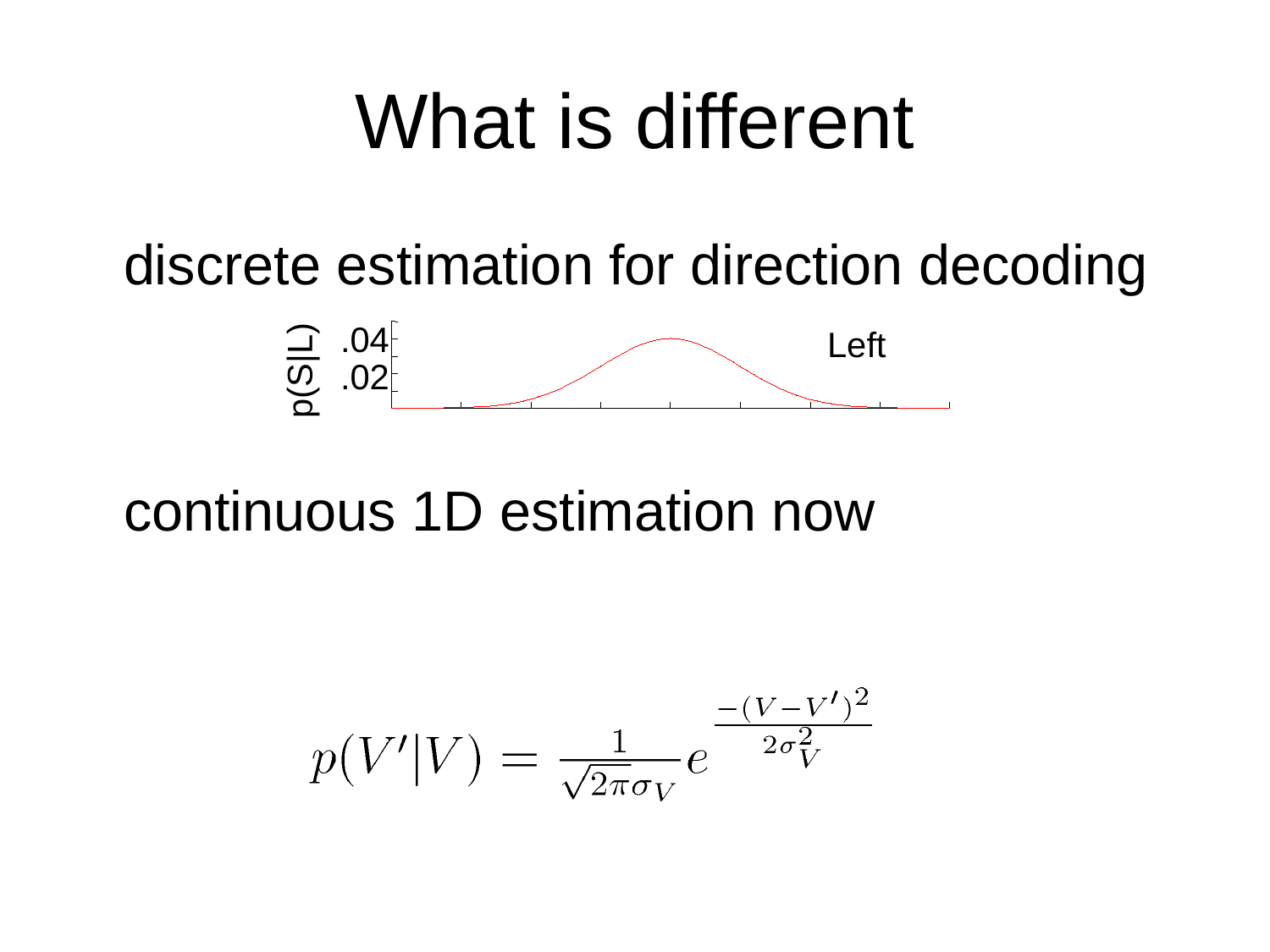

# What is different
discrete estimation for direction decoding
continuous 1D estimation now
.04
Left
p(S|L)
.02

## Slide 33
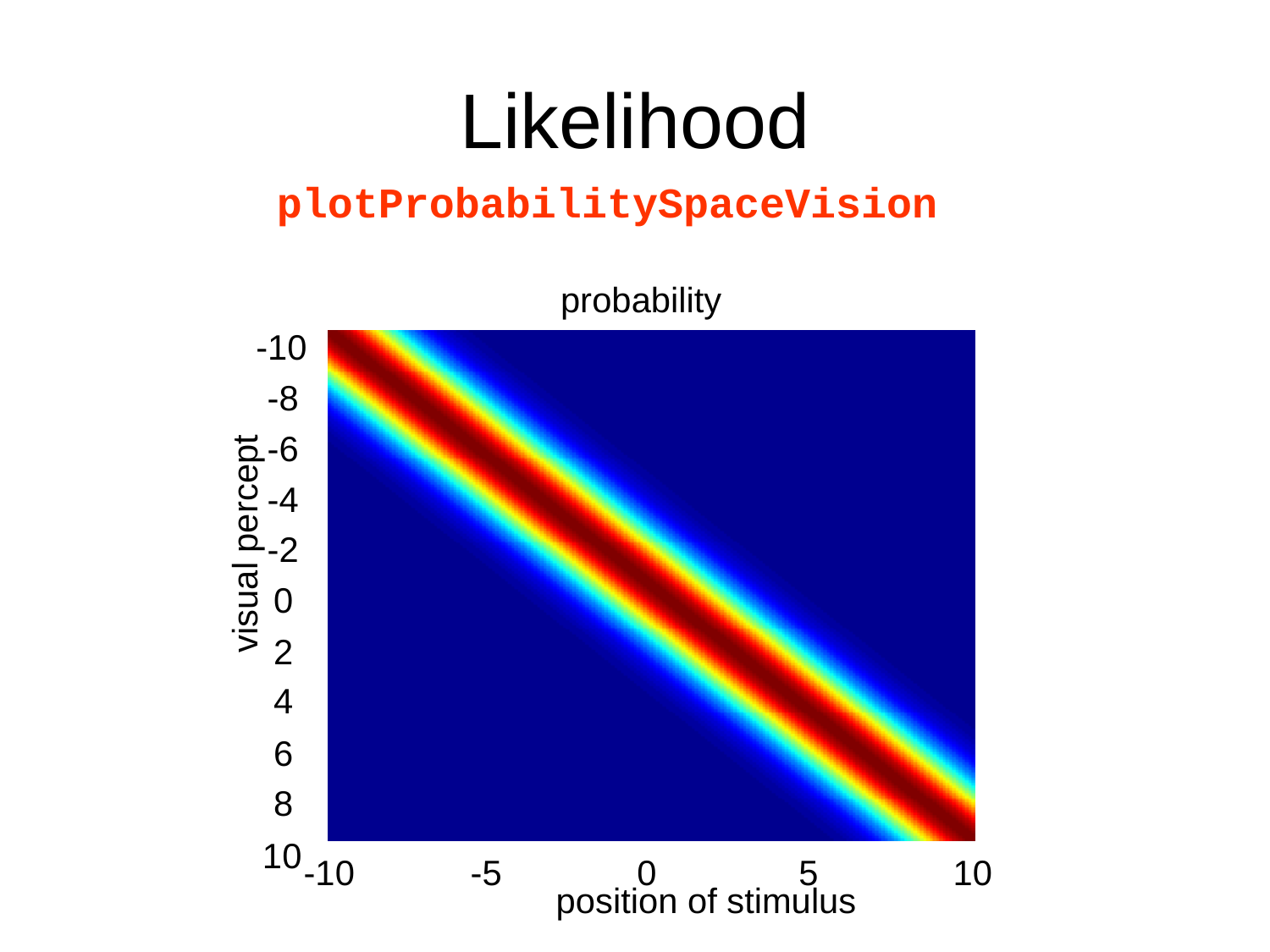

# Likelihood
plotProbabilitySpaceVision
probability
-10
-8
-6
-4
visual percept
-2
0
2
4
6
8
10
-10
-5
0
5
10
position of stimulus

## Slide 34
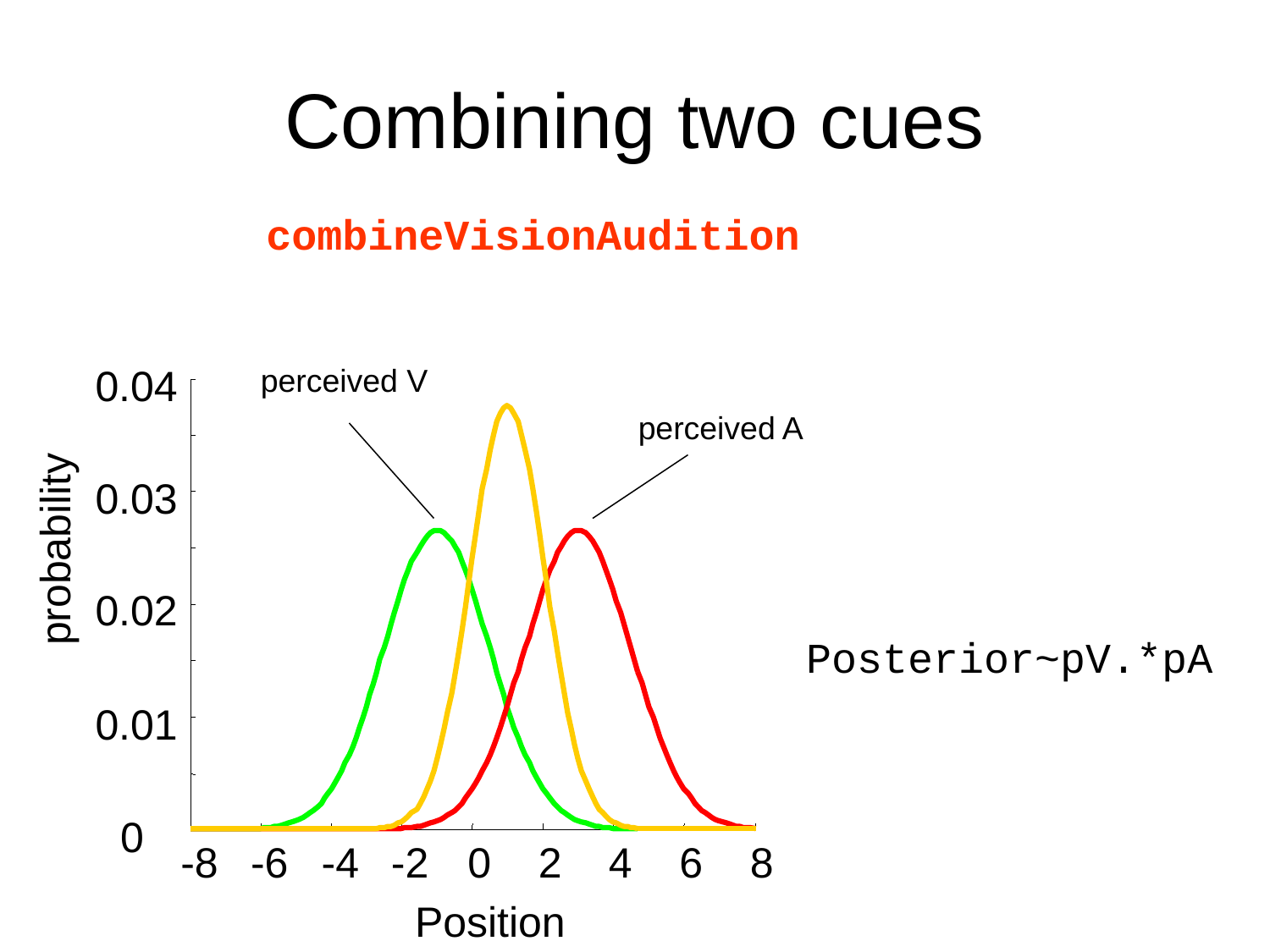

# Combining two cues
combineVisionAudition
perceived V
0.04
perceived A
0.03
probability
0.02
Posterior~pV.*pA
0.01
0
-8
-6
-4
-2
0
2
4
6
8
Position

## Slide 35
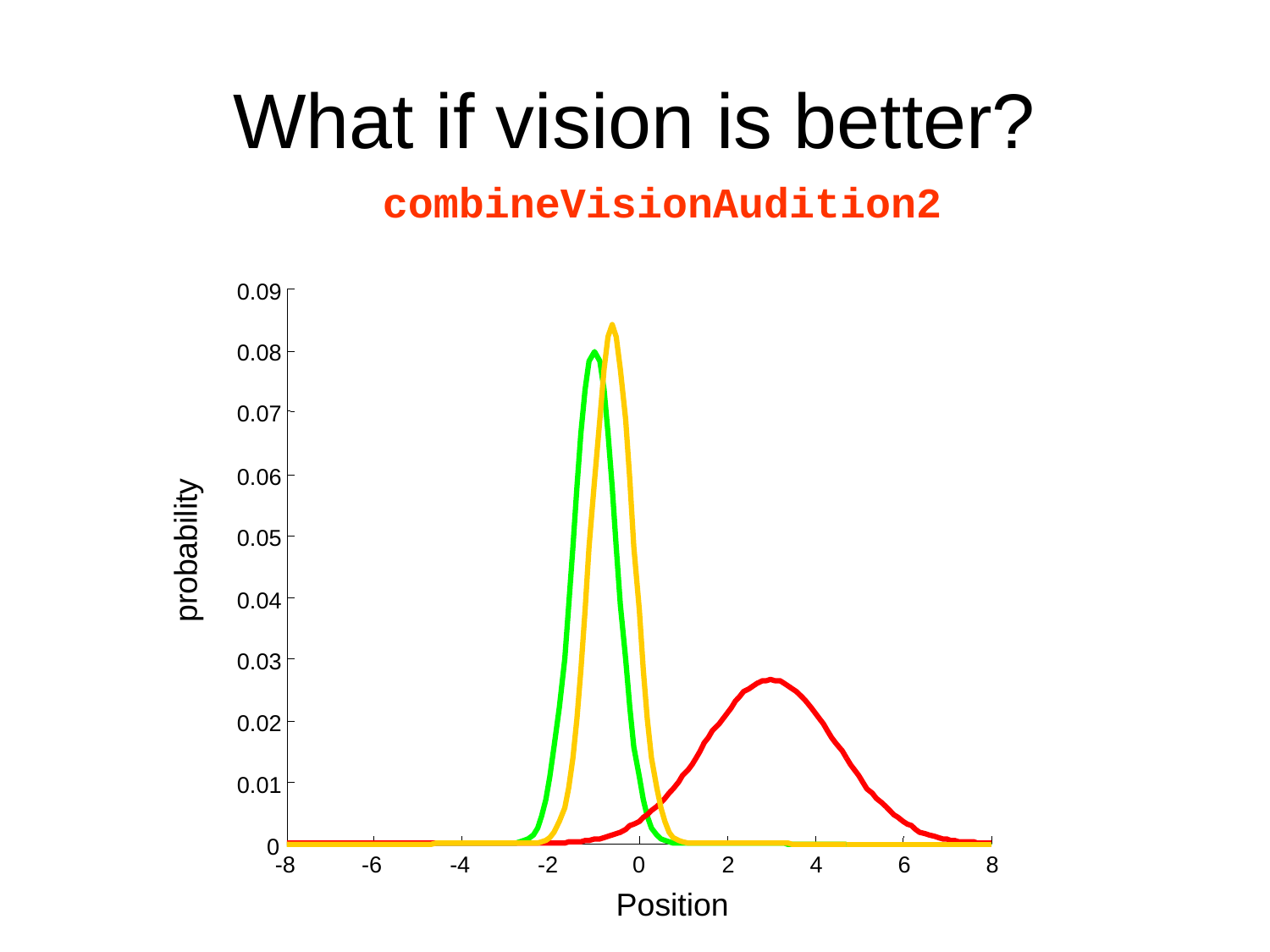

# What if vision is better?
combineVisionAudition2
0.09
0.08
0.07
0.06
0.05
probability
0.04
0.03
0.02
0.01
0
-8
-6
-4
-2
0
2
4
6
8
Position

## Slide 36
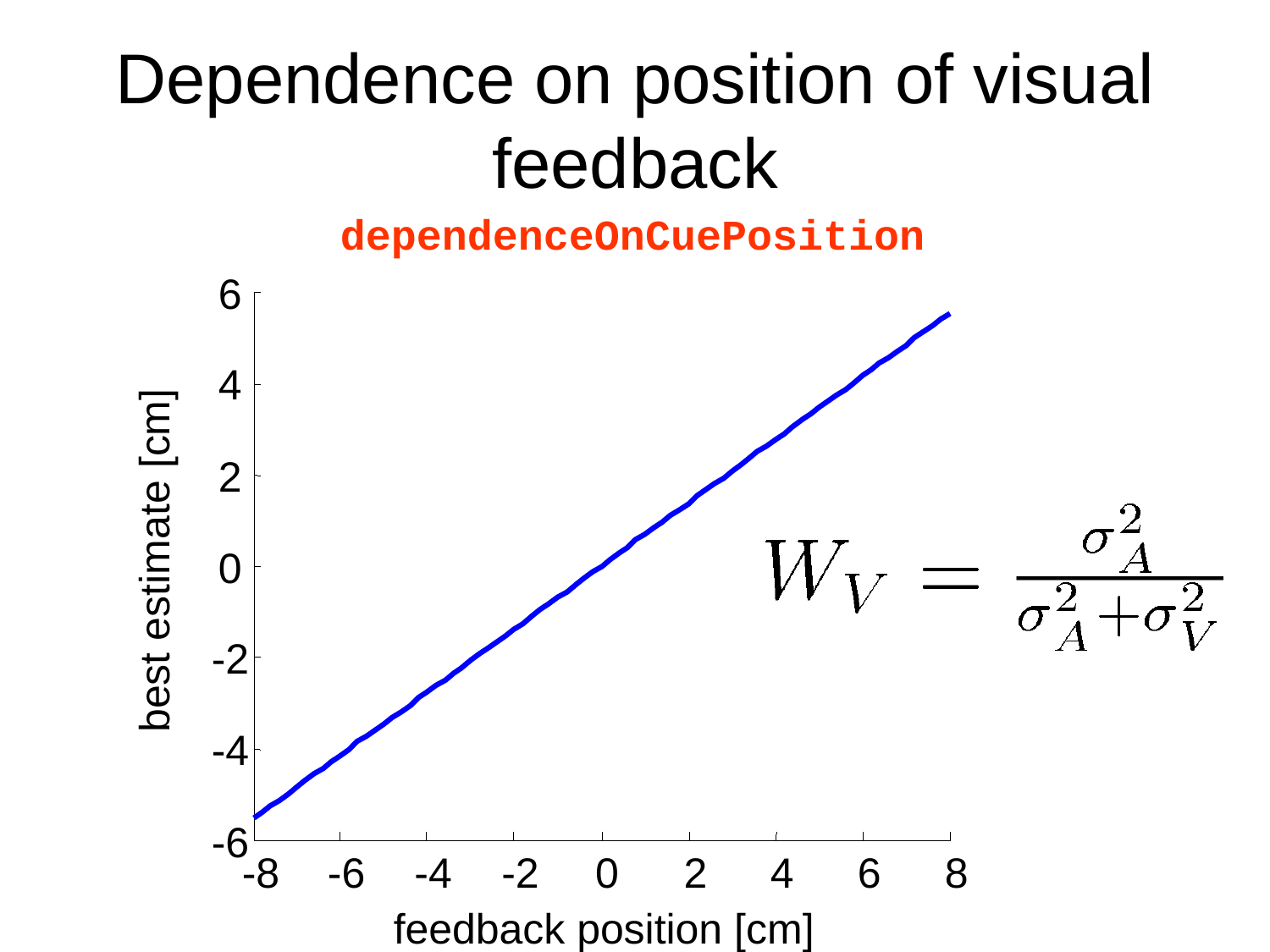

# Dependence on position of visual feedback
dependenceOnCuePosition
6
4
2
best estimate [cm]
0
-2
-4
-6
-8
-6
-4
-2
0
2
4
6
8
feedback position [cm]

## Slide 37
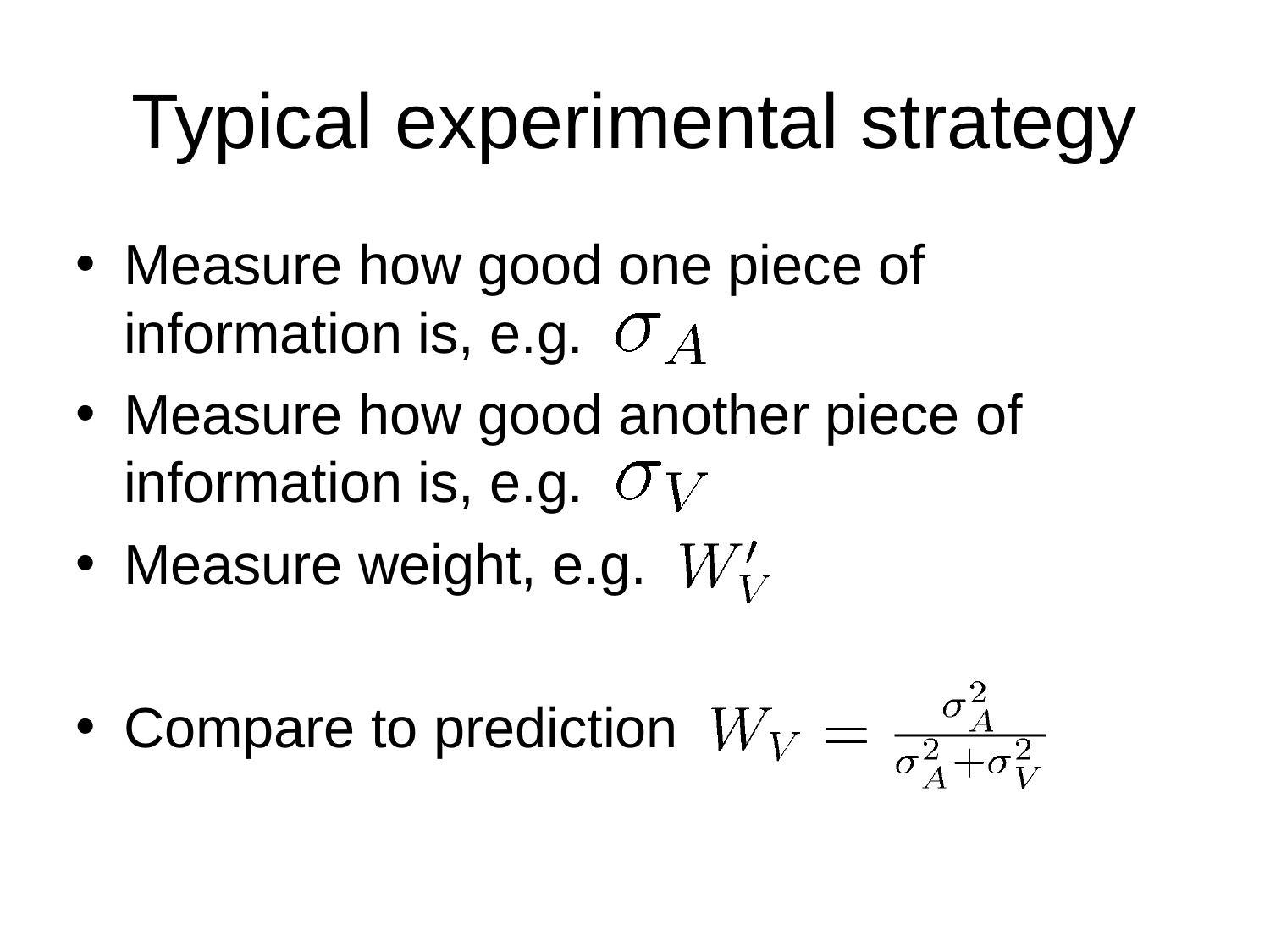

# Typical experimental strategy
Measure how good one piece of information is, e.g.
Measure how good another piece of information is, e.g.
Measure weight, e.g.
Compare to prediction

## Slide 38
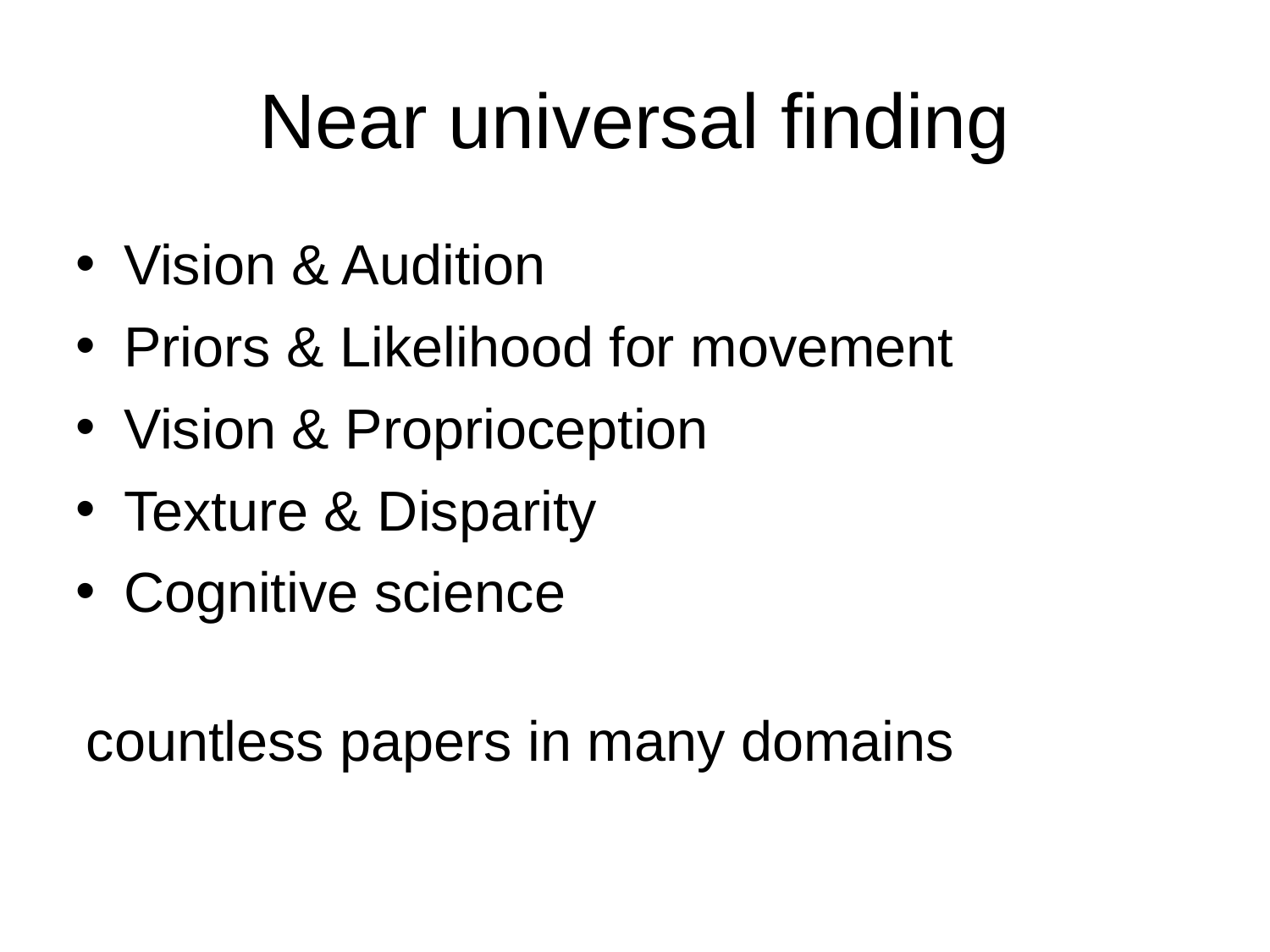

# Near universal finding
Vision & Audition
Priors & Likelihood for movement
Vision & Proprioception
Texture & Disparity
Cognitive science
countless papers in many domains

## Slide 39
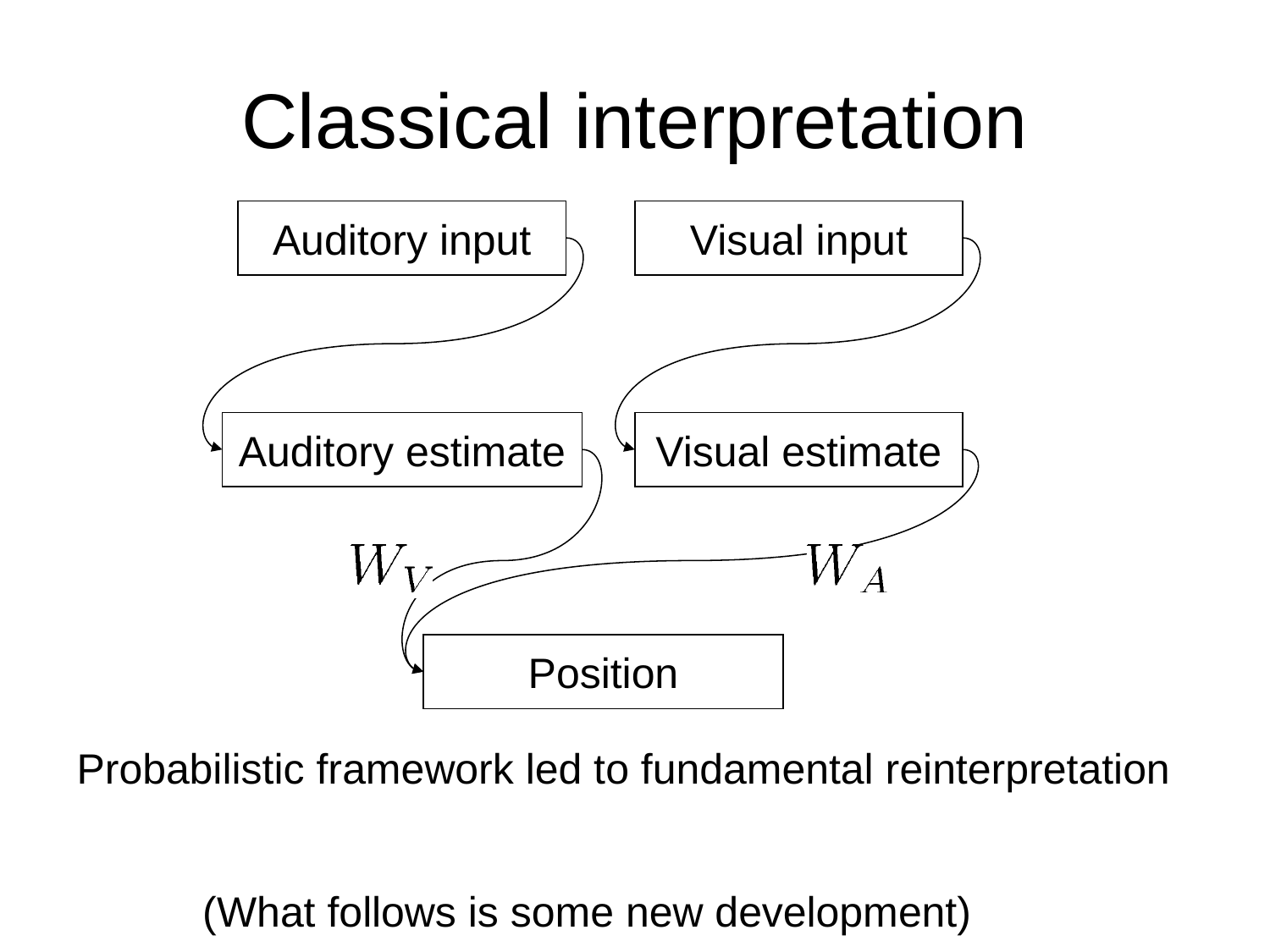

# Classical interpretation
Auditory input
Visual input
Auditory estimate
Visual estimate
Position
Probabilistic framework led to fundamental reinterpretation
(What follows is some new development)

## Slide 40
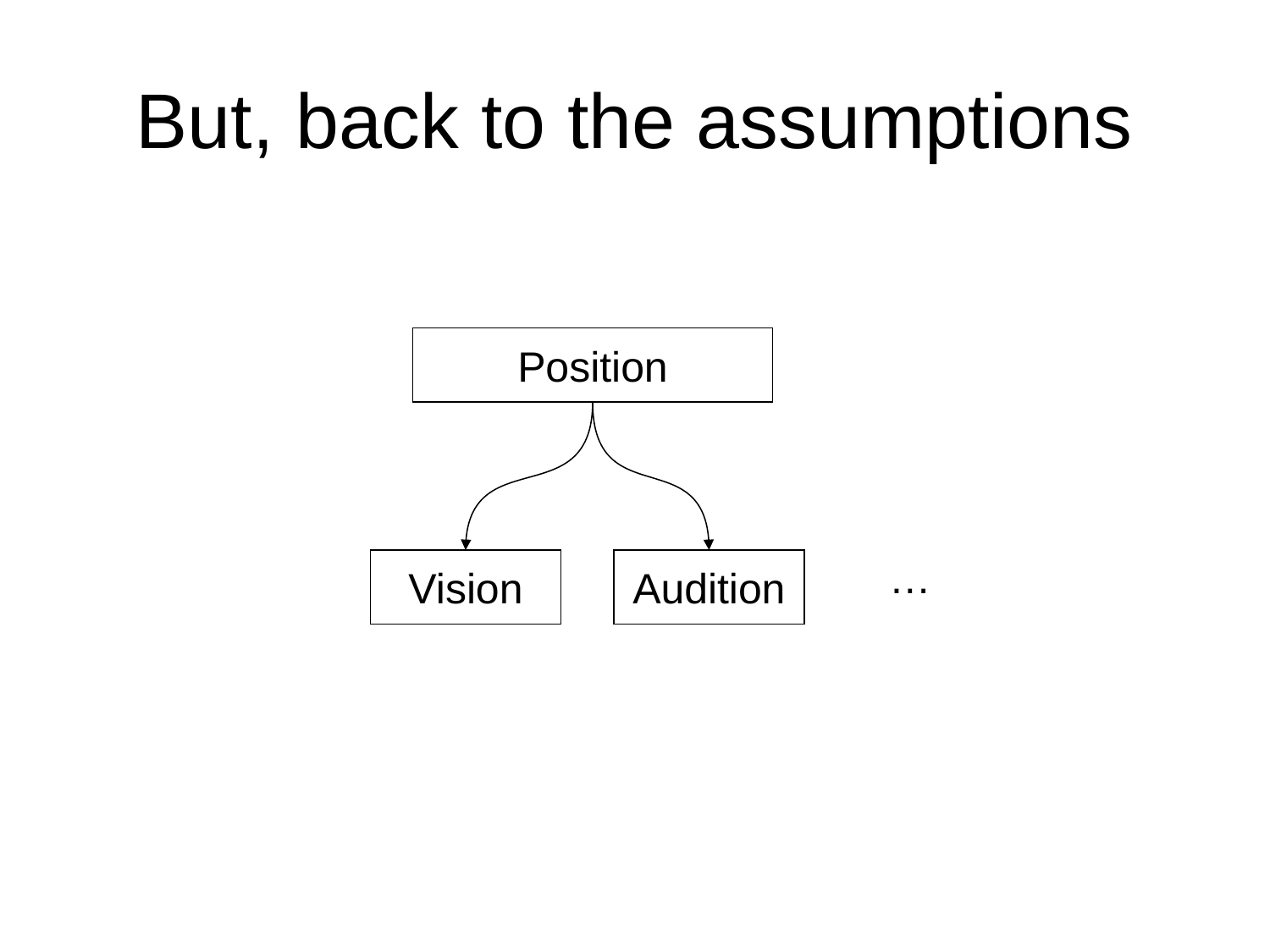

# But, back to the assumptions
Position
…
…
Vision
Audition

## Slide 41
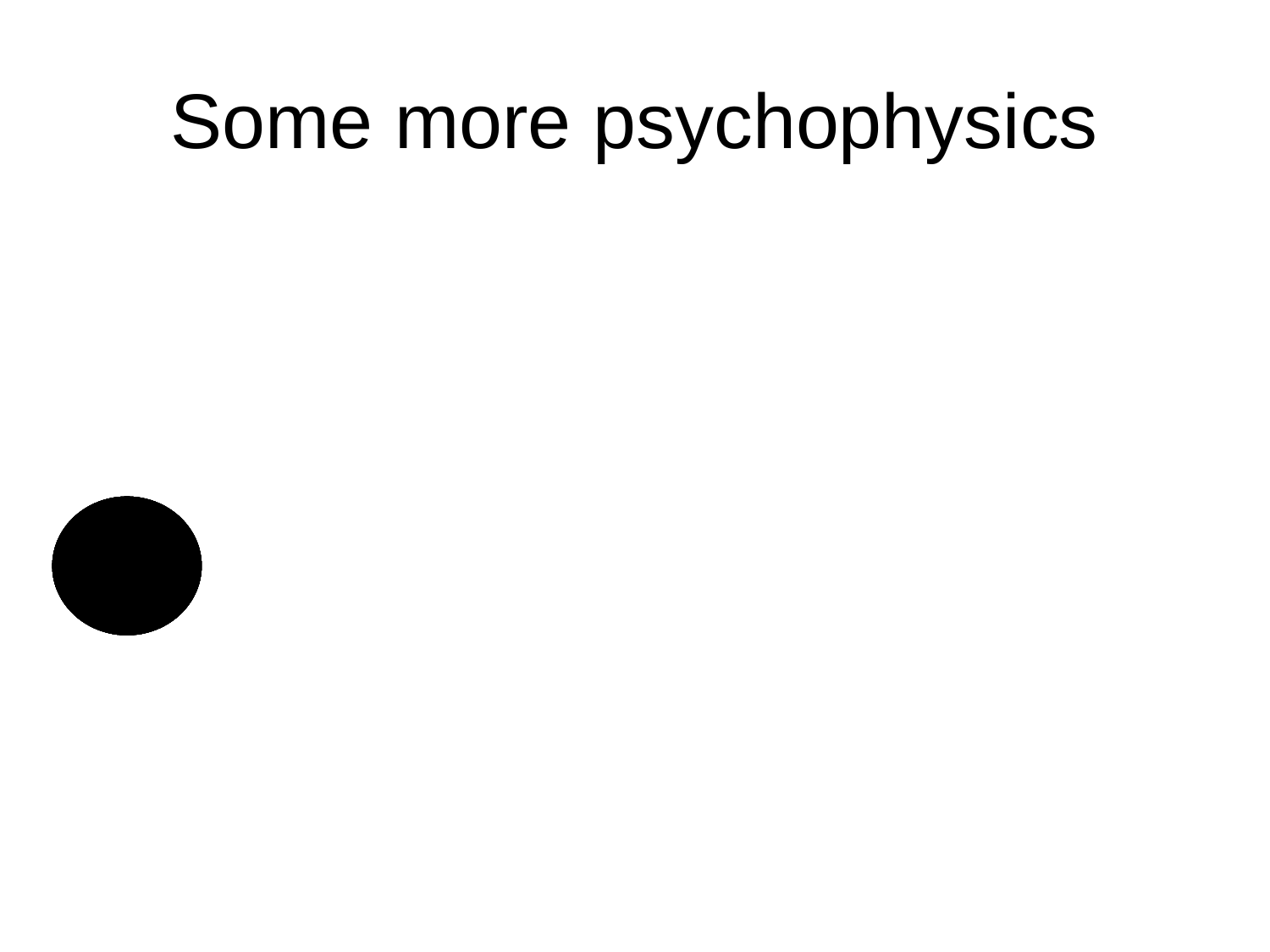

# Some more psychophysics

## Slide 42
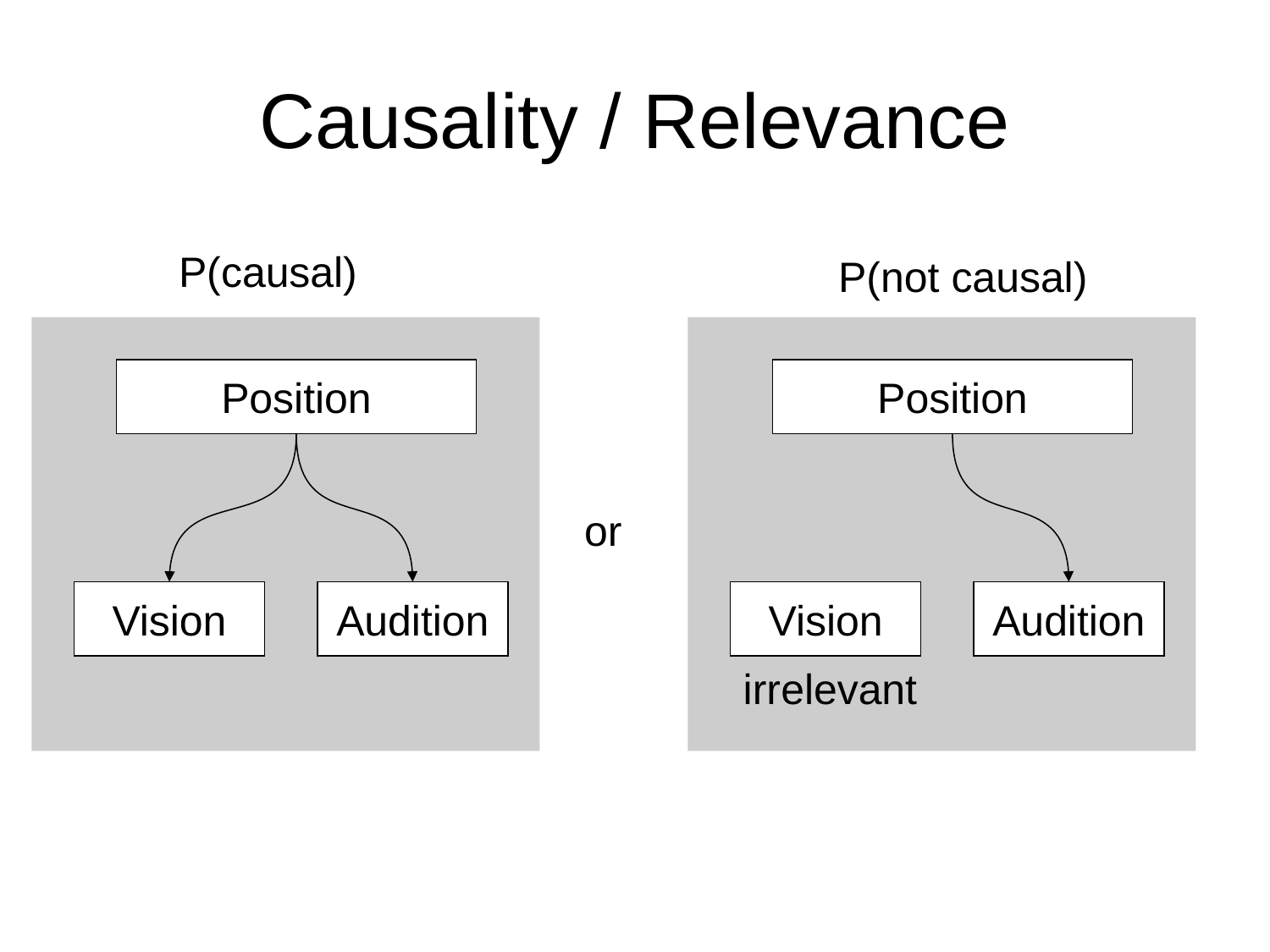

# Causality / Relevance
P(causal)
P(not causal)
Position
Position
or
Vision
Audition
Vision
Audition
irrelevant

## Slide 43
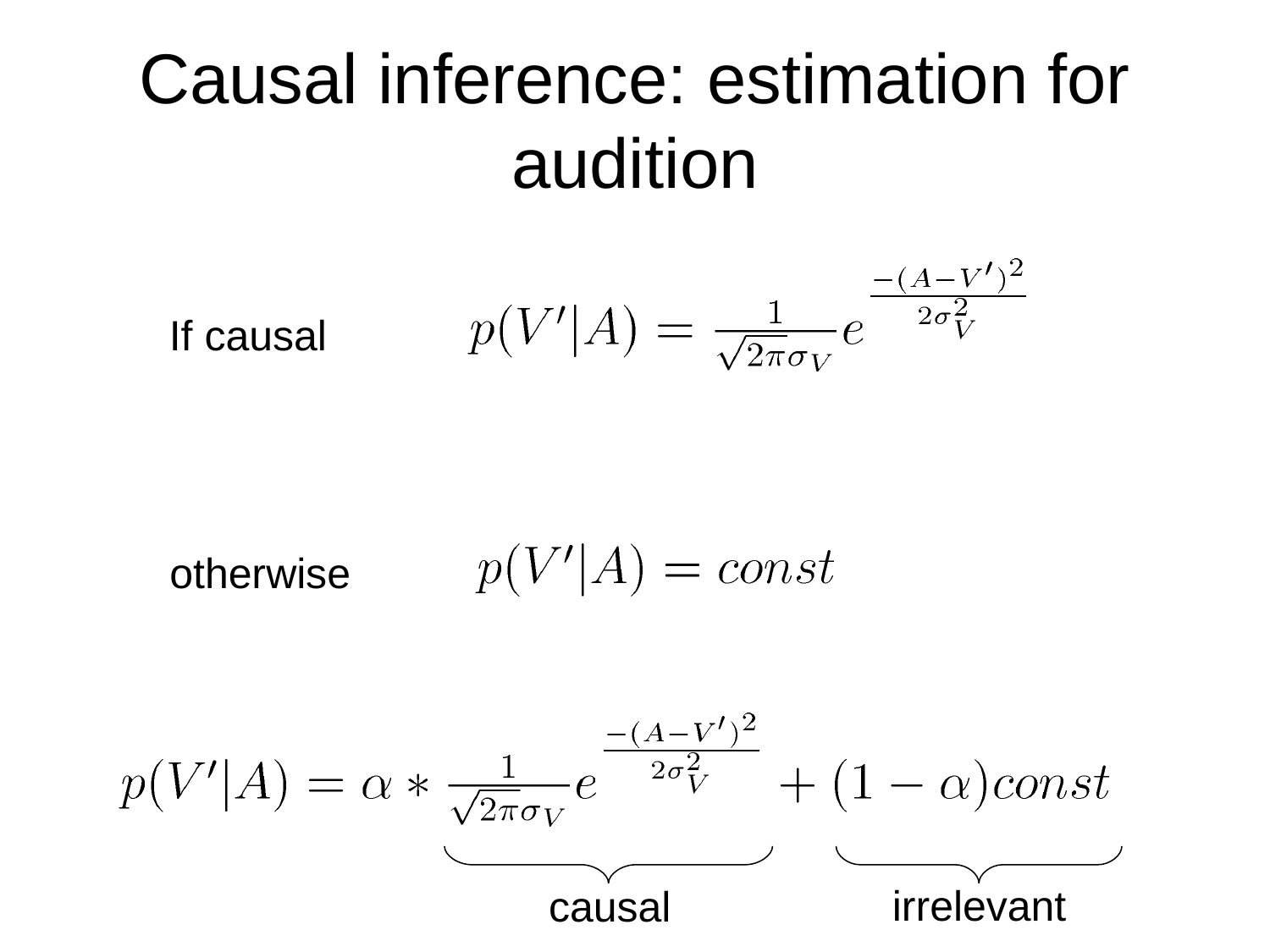

# Causal inference: estimation for audition
If causal
otherwise
irrelevant
causal

## Slide 44
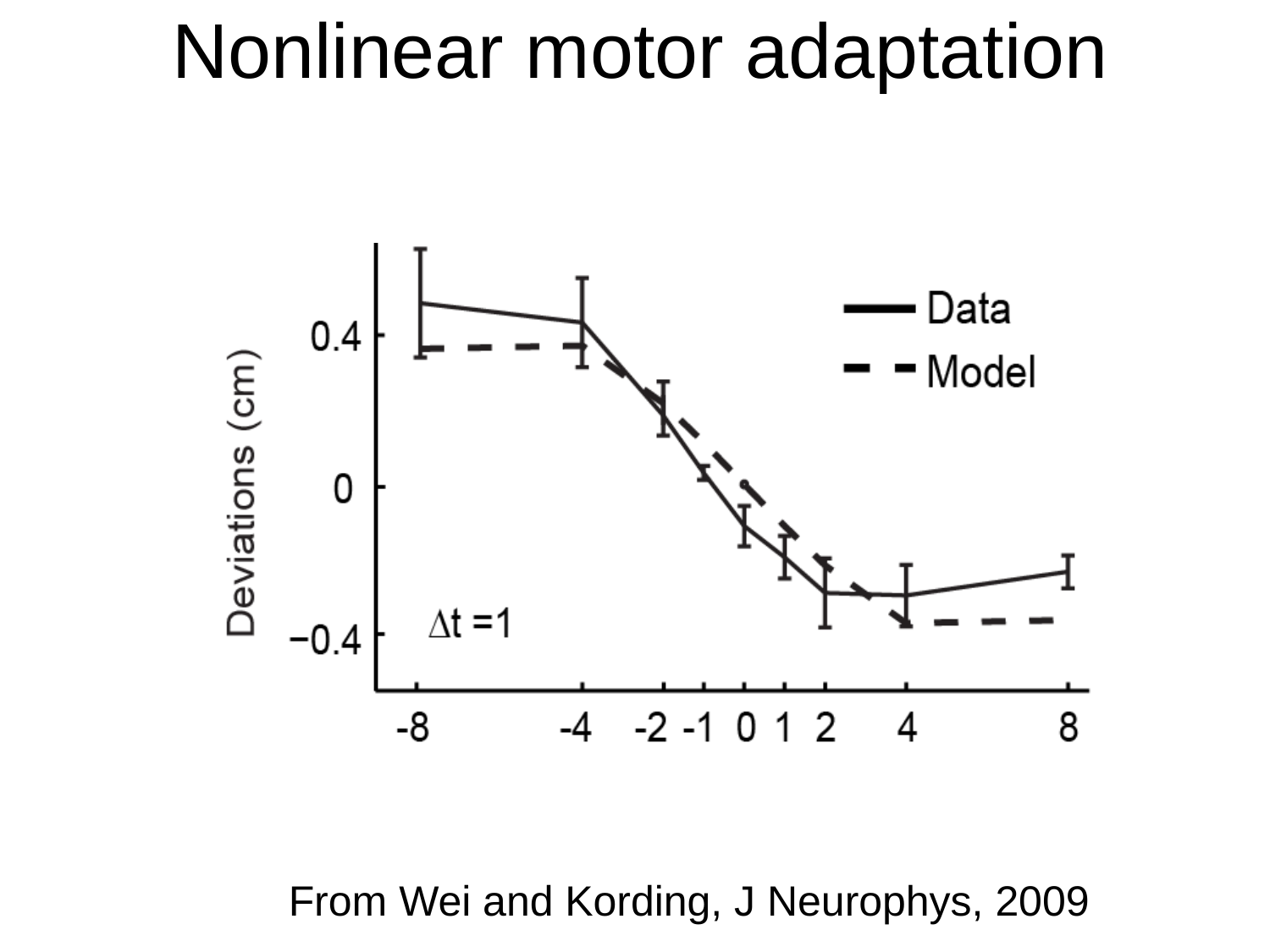

# Nonlinear motor adaptation
From Wei and Kording, J Neurophys, 2009

## Slide 45
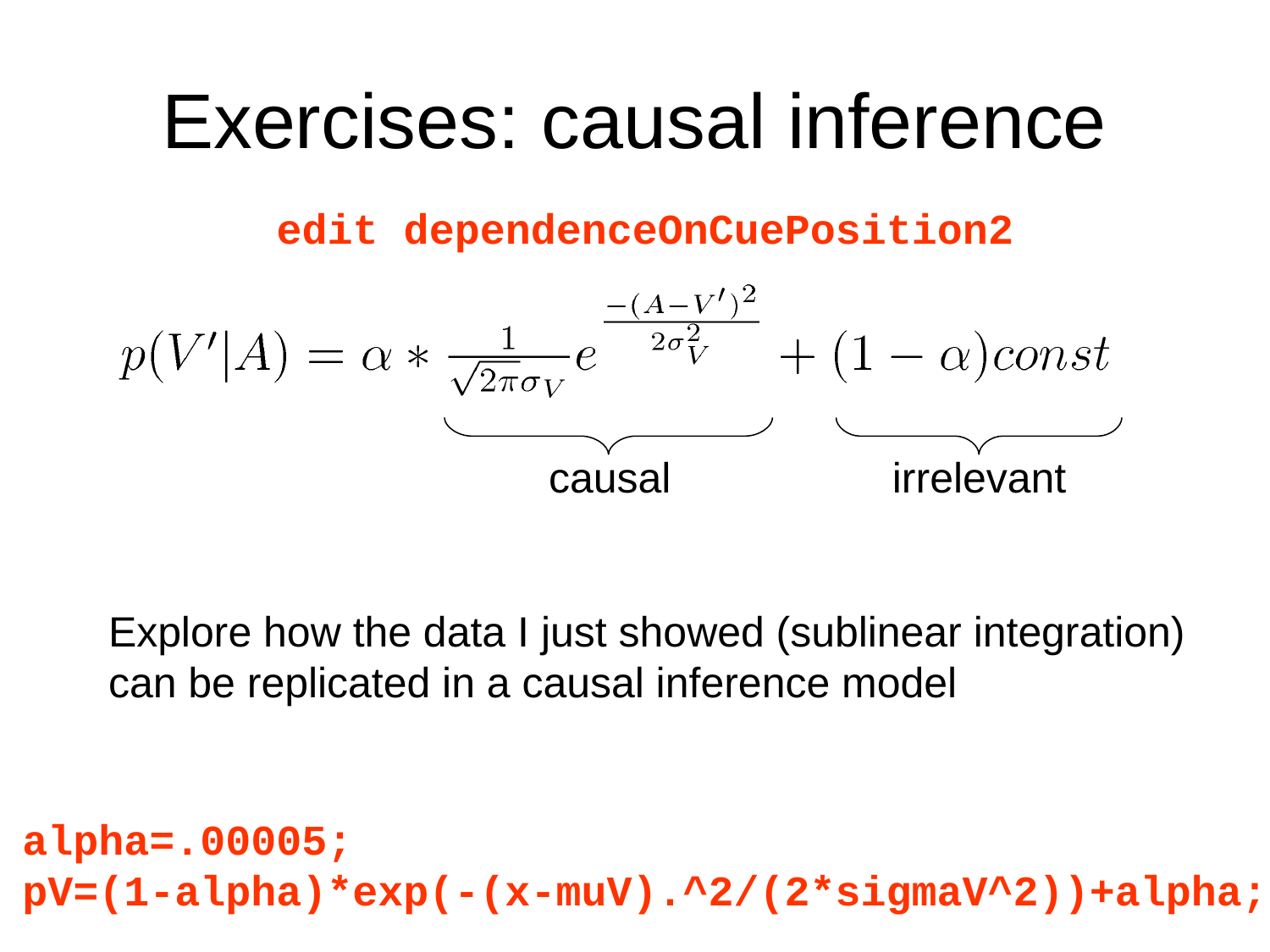

# Exercises: causal inference
edit dependenceOnCuePosition2
irrelevant
causal
Explore how the data I just showed (sublinear integration)
can be replicated in a causal inference model
alpha=.00005;
pV=(1-alpha)*exp(-(x-muV).^2/(2*sigmaV^2))+alpha;

## Slide 46
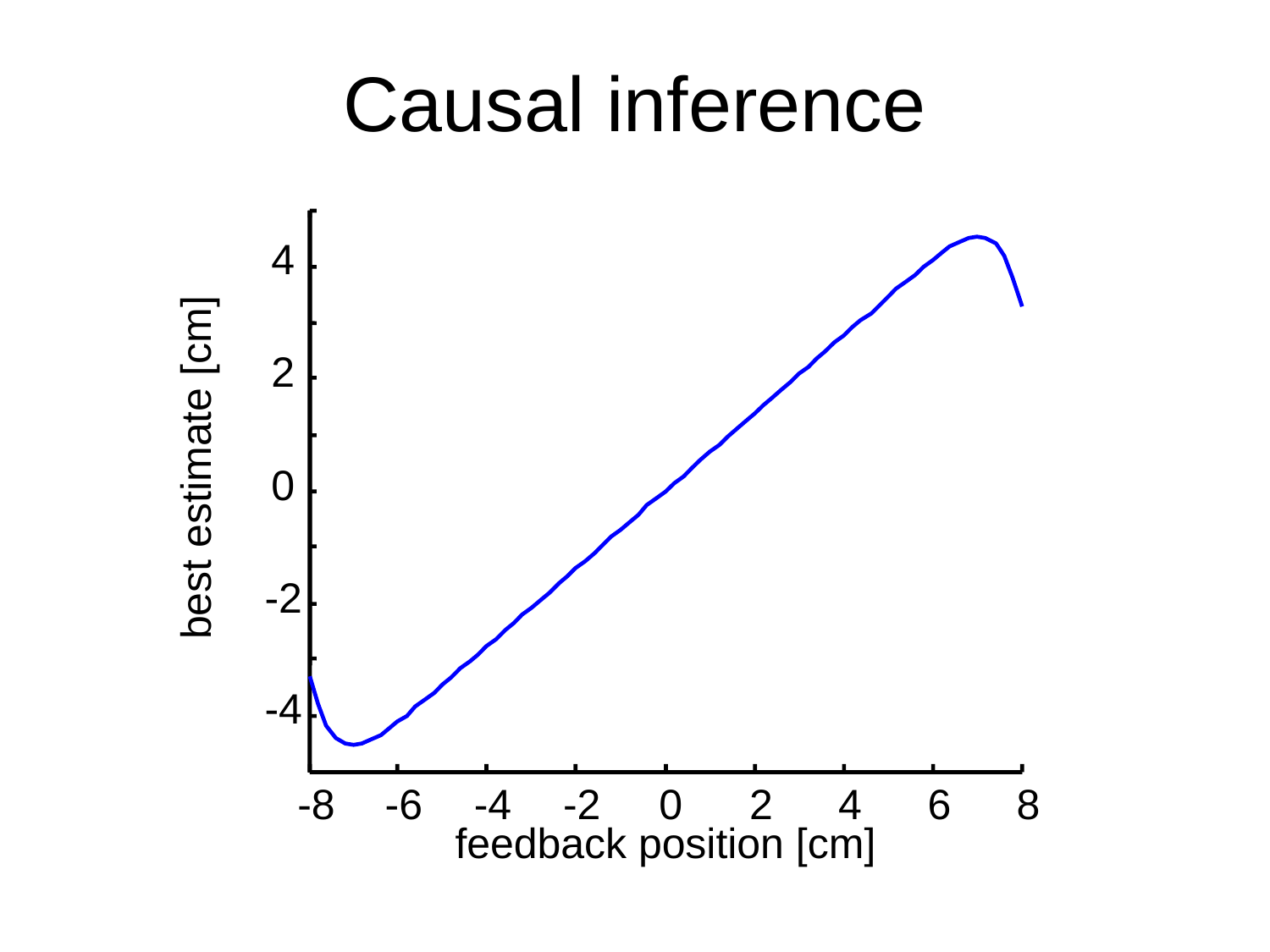

# Causal inference
4
2
best estimate [cm]
0
-2
-4
-8
-6
-4
-2
0
2
4
6
8
feedback position [cm]

## Slide 47
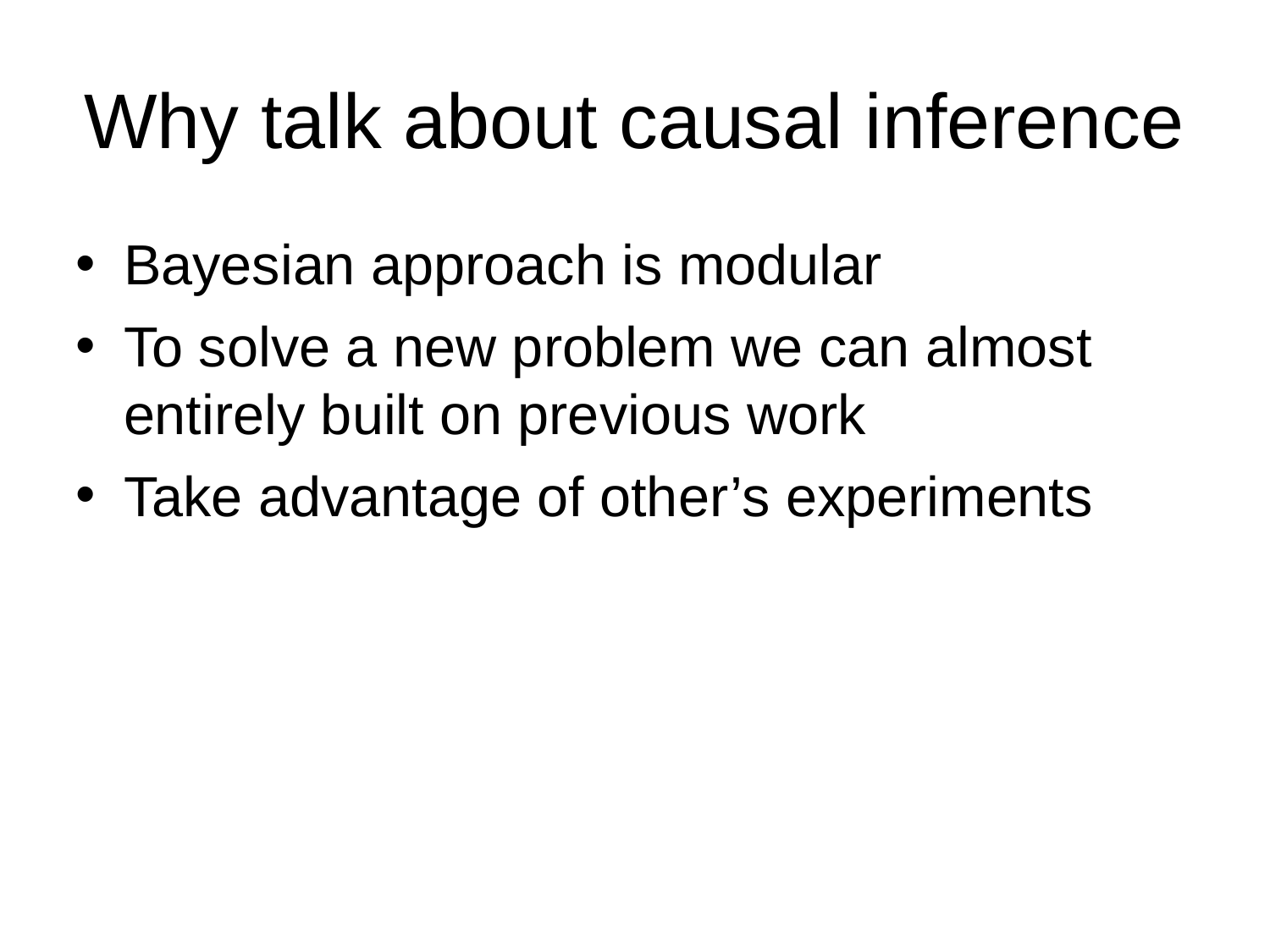

# Why talk about causal inference
Bayesian approach is modular
To solve a new problem we can almost entirely built on previous work
Take advantage of other’s experiments

## Slide 48
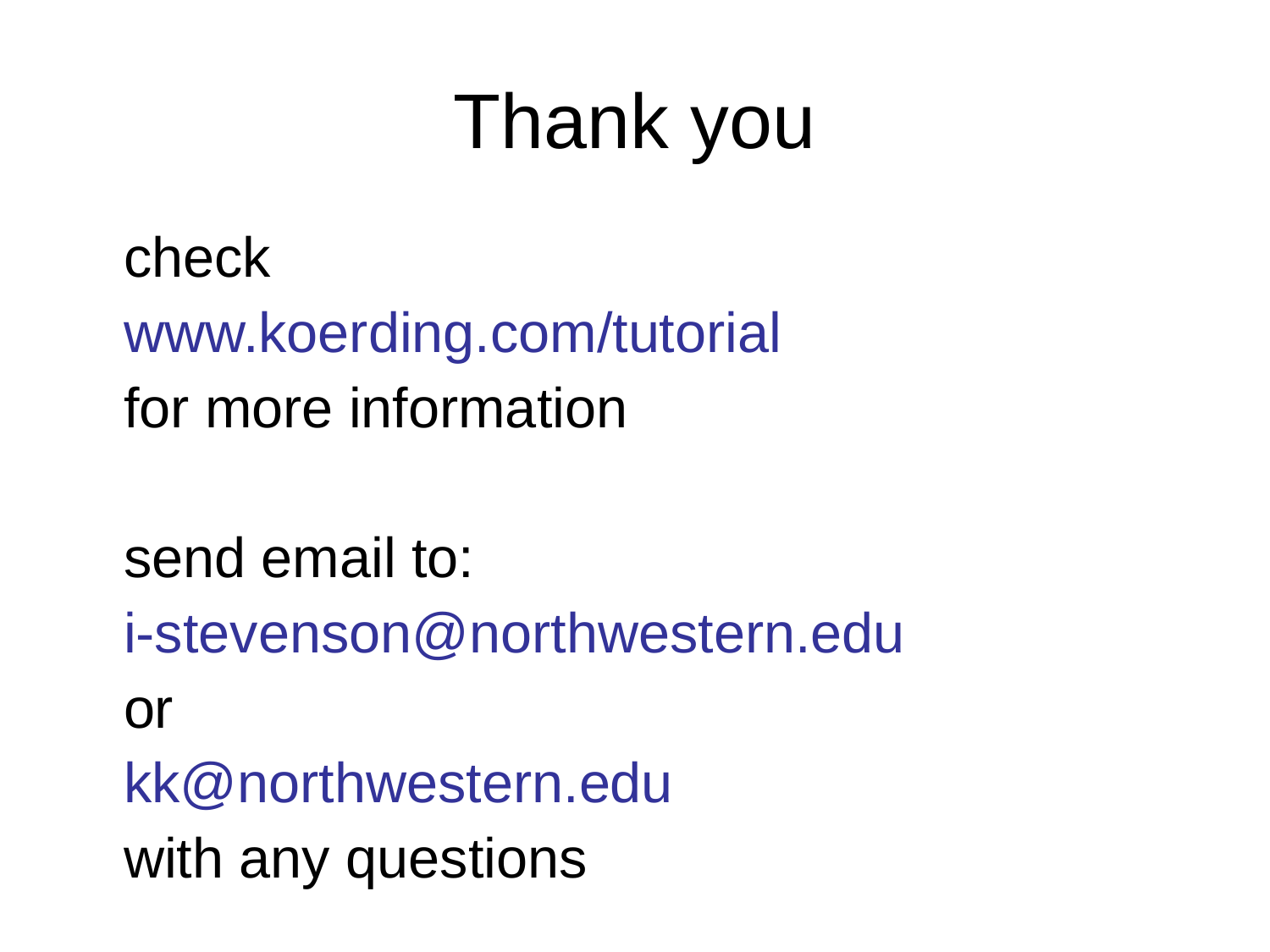

# Thank you
check
www.koerding.com/tutorial
for more information
send email to:
i-stevenson@northwestern.edu
or
kk@northwestern.edu
with any questions
