# Supplementary material for: Pain: A Statistical Account
Source: PLoS Comput Biol. 2017 Jan 12;13(1):e1005142. doi: 10.1371/journal.pcbi.1005142 (PMC5230746; doi:10.1371/journal.pcbi.1005142)
Supplement: S2 File — (DOC) [file pcbi.1005142.s002.doc]

**Bayes Tutorial**

**List of all necessary MATLAB commands for the tutorial:**

**Basic**

*load filename*: loads all the variables from the file with the name filename

*x=a:b:c* produces a vector which starts with a and goes to c with increments of b. For example x=1:0.2:2 produces the vector [1 1.2 1.4 1.6 1.8 2.0]

*[a b]*: Produces a new vector or matrix by pasting together two others

/, *: Division and Multiplication

./,.*: Pointwise division or multiplication. E.g. [1 2 3].*[1 2 3]=[1 4 9]

%: Anything following the % are just comments

**Plotting**

*figure*: opens a new figure

*plot(x,y):* Plots vector y as a function of vector x

*hold on*: Makes it so that the figure is not redrawn when new data is plotted

*hold off*: Makes it so that the figure is again redrawn

*hist(data,pos)*: Plots the histogram of data at the positions centered on pos

*xlabel, ylabel:* Adding labels

**Analysis**

*h=hist(data,pos)*: Stores this histogram into variable h

*mean(data):* mean of a vector or matrix

*std(data):* standard deviation of vector or matrix

*sum(data):* sum

**Functions**

*exp(data):* exponential function

*sqrt(data):* square root

Throughout the tutorial matlab code is written in courier:

ls

for example displays whats in the current directory

**A lot of relevant math**

Relevant math for problems/exercises will be given on the slides during the presentation.

***Basic probability rules***

Bayes’ Rule

Definition of joint probability

Definition of marginalization

… a particularly useful case

*Likelihood:*

*Prior:*

*Posterior:*

*Gaussian Distribution:*

***Naïve Bayes Classification***

For classifying spike data (S) to determine whether a monkey is moving left or right (L or R) we use Bayes’ Rule

For Naïve Bayes classification we assume that the likelihood is a Gaussian. Using marginalization we can rewrite the denominator

If we have many neurons and if we assume that the neurons are all independent

***Cue Combination***

Given two pieces of noisy information, say visual and auditory cues, what’s the best way to combine them? Assume they’re both Gaussian…

If we assume the cues are independent the combination is simply a product of Gaussians

which has the analytic solution

**Worksheet (commands to be used):**

**Part I: Decoding movement intent from firing patterns**

**Command What it does**

load datasetStart loads simulated 2Neuron dataset

plotHistograms plots hist of right and left moves

plotProbability converts histograms into probability

plotProbabilitiesFromGauss replot probability from mean and std

decodeOneNeuron optimal decoding using one neuron

**Exercise 1:** In this exercise you combine info

edit combineTwoNeurons from two neurons using math

realData Display the data from real neurons

(thanks to Lee Miller + lab)

realNeuronDecoding Combine info from 52 neurons to

decode movement

**Part I: Modeling Human Behavior, Cue Combination**

plotProbabilitySpaceVision Plot likelihood function

combineVisionAudition Use 2 cues to estimate target

combineVisionAudition2 same but with better vision

dependenceOnCuePosition Analyze influence of cue on estimate

**Exercise 2:**

edit dependenceOnCuePosition2 Analyze effect of “outliers” or irrelevant information
